# Alterations of the human gut microbiome in multiple sclerosis

**DOI:** 10.1038/ncomms12015

**Published:** 2016-06-28

**Authors:** Sushrut Jangi, Roopali Gandhi, Laura M. Cox, Ning Li, Felipe von Glehn, Raymond Yan, Bonny Patel, Maria Antonietta Mazzola, Shirong Liu, Bonnie L. Glanz, Sandra Cook, Stephanie Tankou, Fiona Stuart, Kirsy Melo, Parham Nejad, Kathleen Smith, Begüm D. Topçuolu, James Holden, Pia Kivisäkk, Tanuja Chitnis, Philip L. De Jager, Francisco J. Quintana, Georg K. Gerber, Lynn Bry, Howard L. Weiner

**Affiliations:** 1 Ann Romney Center for Neurologic Diseases, Evergrande Center for Immunologic Diseases, Partners Multiple Sclerosis Center, Brigham and Women's Hospital, Department of Neurology, Harvard Medical School, Boston, Massachusetts 02115, USA; 2 Center for Clinical and Translational Metagenomics, Department of Pathology, Brigham and Women's Hospital, Harvard Medical School, Boston, Massachusetts 02115, USA; 3 Department of Microbiology, University of Massachusetts, Amherst, Massachusetts 01003, USA

## Abstract

The gut microbiome plays an important role in immune function and has been implicated in several autoimmune disorders. Here we use 16S rRNA sequencing to investigate the gut microbiome in subjects with multiple sclerosis (MS, *n*=60) and healthy controls (*n*=43). Microbiome alterations in MS include increases in *Methanobrevibacter* and *Akkermansia* and decreases in *Butyricimonas*, and correlate with variations in the expression of genes involved in dendritic cell maturation, interferon signalling and NF-kB signalling pathways in circulating T cells and monocytes. Patients on disease-modifying treatment show increased abundances of *Prevotella* and *Sutterella*, and decreased *Sarcina*, compared with untreated patients. MS patients of a second cohort show elevated breath methane compared with controls, consistent with our observation of increased gut *Methanobrevibacter* in MS in the first cohort. Further study is required to assess whether the observed alterations in the gut microbiome play a role in, or are a consequence of, MS pathogenesis.

## Abstract (web summary)

The gut microbiome has been implicated in several autoimmune disorders. Here, the authors study the gut microbiome of patients with multiple sclerosis, and find correlations between altered abundance of certain gut microorganisms and changes in expression of immune defence genes.

## Graphical abstract

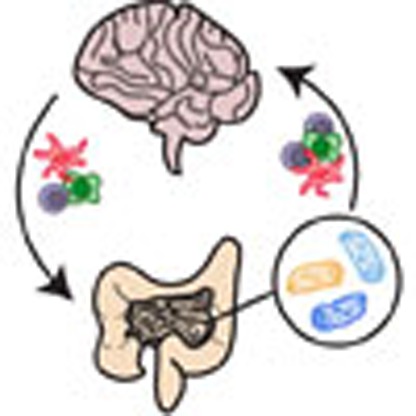

Microorganisms in the human gut encompass hundreds to thousands of bacterial, archaeal, viral and fungal species, making the human intestinal lumen a rich and dense source of antigenic diversity^1^. The gut mucosal immune system samples and processes these microbial antigens, potentially driving the expansion of particular immune subsets or generating specific immune repertoires^2^. Thus, the intestinal microbiome is an important entity within the host that influences immune responses both locally and systemically.

The gut microbiome has been implicated in numerous immunologic disorders, including multiple sclerosis (MS), inflammatory bowel disease, type 1 diabetes and rheumatoid arthritis^3^,^4^,^5^. In experimental autoimmune encephalomyelitis (EAE), a murine model for MS, altering the gut microbiome modulates central nervous system (CNS) autoimmunity. In a relapsing–remitting mouse model of spontaneous EAE, transgenic SJL/J mice raised in germ-free conditions were protected against developing the disease, while the introduction of commensal microbiota into the gut restored susceptibility^6^. While gnotobiotic mice are relatively immunocompromised due to lack of microbial stimulation promoting immune maturation, specific association of germ-free mice with defined commensal species has been shown to modulate the development and severity of EAE. Segmented-filamentous bacteria (SFB) drive expansion of Th17 cell populations and generation of interleukin (IL)-17 in the gut^7^. Mono-colonization of the gut of C57BL/6 mice with segmented-filamentous bacteria promotes Th17 accumulation in the spinal cords of mice and induces the development of EAE^8^. Conversely, treatment of C57BL/6 mice with a polysaccharide from the organism *Bacteroides fragilis* expands intestinal Foxp3+ CD4 Tregs and protects against the development of CNS autoimmunity^9^,^10^.

In the case of human autoimmune disease, associations have been reported with different members of the commensal microbiota. In a study of 20 MS patients versus 40 healthy controls, *Faecalibacterium*, *Prevotella* and *Anaerostipes* were decreased in MS, but the connection between microbiota, treatment and changes in immunity was not examined^11^. *Prevotella copri* has been associated in proinflammatory conditions, and has found to be enriched in patients with new-onset rheumatoid arthritis^5^, or capable of exacerbating dextran sodium sulfate colitis in antibiotic-treated C57BL/6 mice. Butyrate-producing organisms have protective associations with inflammatory conditions, for example, *Faecalibacterium prausnitzii* has been shown to be reduced in inflammatory bowel disease^12^. In neuromyelitis optica, a CNS autoimmune disease directed against aquaporin-4, there are increased antibodies against gastrointestinal antigens and cross-reactivity to a protein belonging to *Clostridium perfringens*, suggesting that autoimmunity in neuromyelitis optica may be driven by molecular mimicry against microbial antigens^13^. Similarly, the autoimmunity associated with Guillain–Barre syndrome has been associated with *Campylobacter jejuni* and the generation of antibodies to microbial components that cross-react with epitopes on the surface of the neuron^14^.

Given the importance of the gut microbiome in immune function and autoimmune disease, for the present work we investigated the human gut microbiome in multiple sclerosis (MS). We identify alterations in the intestinal microbiota and find correlations with MS-associated immune changes and treatment. If further studies demonstrate that these candidate microorganisms play an active role in either contributing to or ameliorating MS, then there is the potential to develop new diagnostics and therapies to combat the disease.

## Results

### Subject characteristics

**Figure 1:**
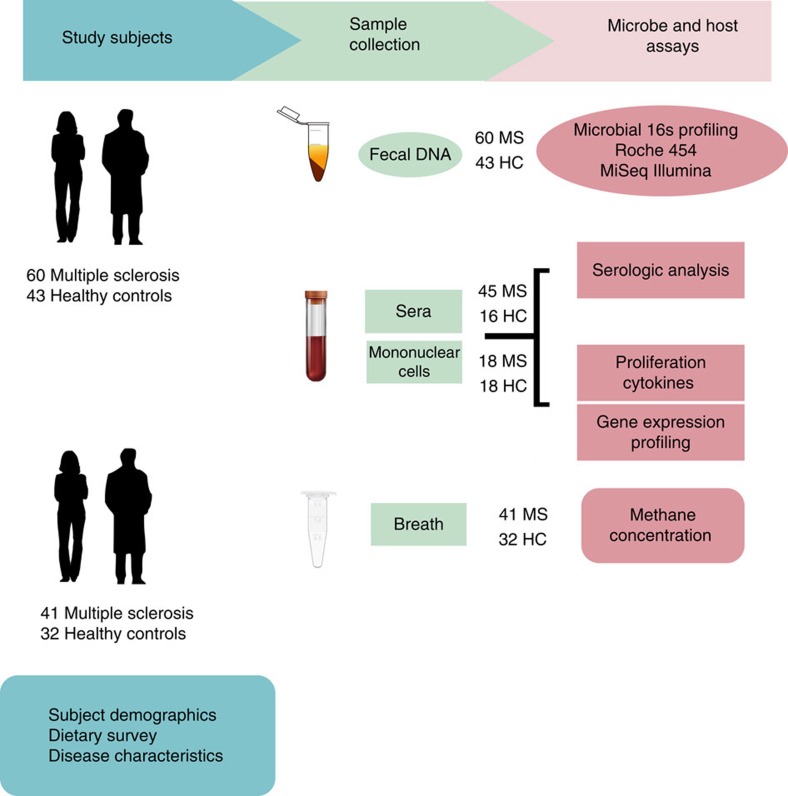
Study design. Faecal samples were collected from MS patients (*n*=60) and healthy subjects (*n*=43). Microbial DNA was extracted from frozen faecal samples and 16s rDNA sequencing was performed using Roche 454 and Illumina platforms. Gene expression profiling was performed on circulating monocytes and T cells from MS patients (*n*=18) and healthy subjects (*n*=18) using a Nanostring platform. Peripheral blood mononuclear cells were collected from MS patients (*n*=18) and healthy subjects (*n*=18) to conduct proliferation and cytokine assays in response to specific microbial stimulation. Sera from MS patients (*n*=45) and healthy subjects (*n*=16) was collected for ELISA-based techniques to capture serologic activity directed against specific microbes. Breath samples from MS patients (*n*=41) and healthy subjects (*n*=32) were collected from a second subject cohort to determine breath methane concentrations.

Faecal samples were collected from 60 MS patients and 43 healthy controls (Fig. 1); details of the study population are provided in Table 1 and in Methods. The MS and control cohorts had comparable demographic characteristics except that the MS cohort had an increased proportion of males. All MS patients had relapsing–remitting disease but none had an active relapse at the time of study enrollment.

### Structure and composition of the gut microbiome in MS

Microbial DNA was extracted from faecal samples and 16S rRNA gene sequencing was performed on the Roche 454 and Illumina MiSeq platforms using primers targeting the V3–5 or the V4 variable regions, respectively. We used two sequencing platforms to avoid platform-specific biases and to provide complementary information: the Roche 454 platform produces longer sequencing reads but fewer reads, whereas the Illumina MiSeq provides shorter reads but greater sequencing depth. The resulting sequences were then processed using the mothur software package for quality filtering, removal of artifacts and clustering to operational taxonomic units (OTUs)^15^ (Supplementary Fig. 1). Roche 454 sequencing yielded 1,426,326 reads of ∼450 nucleotides each, with 426 OTUs identified after quality filtering. Illumina MiSeq sequencing yielded 11,498,168 paired-end reads of 150 nucleotides each, with 1,191 OTUs identified after filtering.

To assess overall differences in microbial community structure in MS patients and controls, we calculated measures of alpha- and beta-diversity. Alpha-diversity summarizes the microbial diversity within each sample, whereas beta diversity measures differences between samples. Shannon entropy, an alpha-diversity measurement of richness and evenness, was measured at multiple sequencing depths using rarefaction curves and was similar between MS patients and healthy controls (Supplementary Fig. 2). To determine whether overall microbial community structure was different between MS patients and controls, we calculated differences in beta-diversity using the weighted and unweighted UniFrac metric. Statistical analyses of the resulting matrices using the analysis of molecular variance technique did not reveal significant differences in overall microbial community structure between the two groups (Supplementary Fig. 3).

### MS-associated microbiota changes at the phylum level

**Figure 2:**
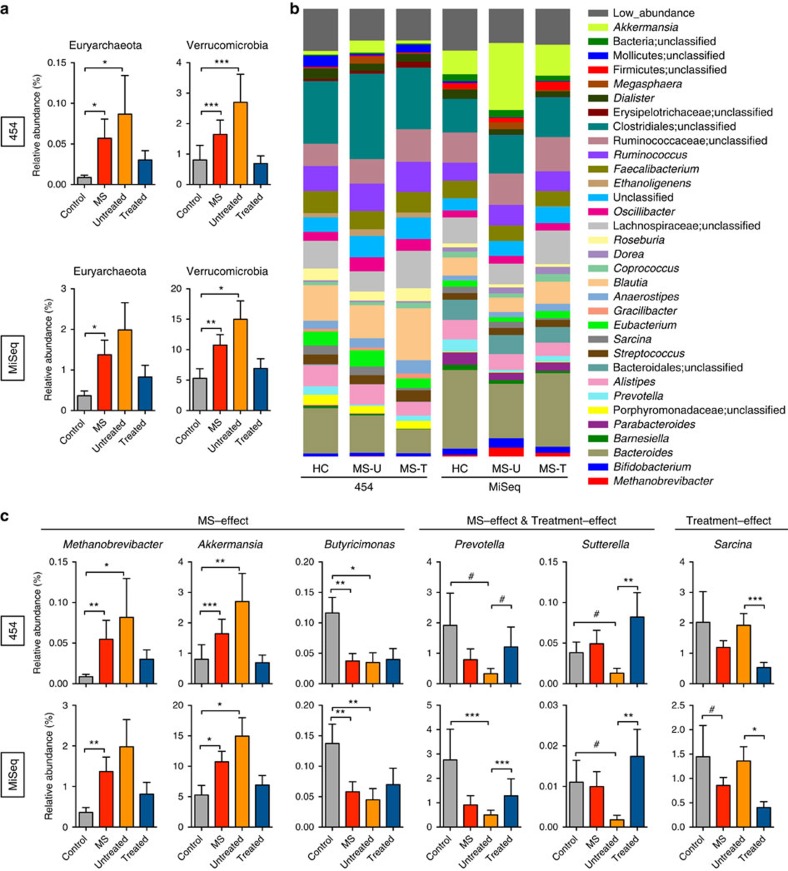
Compositional differences in faecal microbiota between MS patients and healthy subjects. (**a**) Relative abundances of Euryarchaeota and Verrucomicrobia in the faecal microbiota of healthy controls (*n*=43, grey bar), all MS patients (*n*=60, red) and both untreated (*n*=28, orange) and treated MS patient (*n*=32, blue) subgroups as analysed by two independent sequencing technologies, 454 (top) or MiSeq (bottom). (**b**) Relative abundance of prevalent microbiota (>1% in any sample group) determined from MiSeq and 454 high-throughput sequencing. (**c**) Relative abundances of genera in the faecal microbiota that are significantly altered between healthy controls (*n*=43) and MS patients (*n*=60; MS-effect) or between untreated (*n*=28) and treated MS patients (*n*=32) (disease effect) as analysed by two independent sequencing technologies. Significance was determined by DESeq and Benjamini–Hochberg corrected *P* values <0.05 with a false discovery rate threshold of 0.1. Bars represent average, and error bars depict s.e.

At the phylum level, the faecal microbiota of both groups was dominated by Firmicutes and Bacteroidetes, with smaller contributions of Euryarchaeota, Verrucomicrobia and Proteobacteria. The relative abundances of microbiota at the phylum level were compared between the entire MS cohort (both treated and untreated) and controls (Fig. 2). MS patients had a significantly increased relative abundance of the phyla Euryarchaeota and Verrucomicrobia compared to healthy controls (DESeq, Benjamini–Hochberg adjusted *P* value<0.05) by Roche 454 and Illumina sequencing (Fig. 2a, Table 2).

Because immunomodulatory therapy may skew microbiota composition, we separately analysed changes in the microbiota in untreated patients. Both Euryarchaeota and Verrucomicrobia were similarly elevated in untreated MS patients compared with controls, although changes in Euryarchaeota were only significant on the 454 platform, and trended in the same direction on the MiSeq platform. Consistent with other microbiota studies in humans, we detected inter-individual variability within control and MS patients. Thus, we provide rank abundance plots to depict phylum level abundances in each subject (Supplementary Fig. 4).

### MS-associated microbiota changes at the genus level

We next investigated whether relative abundances of the microbiota differed between MS (untreated and treated) patients and controls at the genus level (Fig. 2b,c). The relative abundances of *Methanobrevibacter*, a genus in the phylum Euryarchaeota, and *Akkermansia*, a genus in the phylum Verrucomicrobia, were both increased in MS patients compared with controls by Roche 454 sequencing and by Illumina sequencing (Fig. 2c, Table 2). Furthermore, *Butyricimonas,* which belongs to the phylum Bacteroidetes had a reduced relative abundance in MS as detected by both sequencing platforms. These changes were similarly detected in untreated MS patients compared with controls (Fig. 2c, Supplementary Fig. 5). In addition *Collinsella* and *Slackia*, both belonging to the phylum Actinobacteria, and *Prevotella*, belonging to the phylum Bacteroidetes was decreased in untreated MS patients as detected by both sequencing platforms (Table 2; Supplementary Fig. 6). In general, the results from the two sequencing platforms were concordant, with 98% of the total microbial abundance composed of shared genera. While we were able to detect some primer bias (for example, higher detection of *Akkermansia* with the MiSeq V4 strategy compared with the 454 V35 strategy) the MS-related changes generally remained the same (Supplementary Fig. 7).

### Effect of therapy on gut microbiota

We then asked whether immunomodulatory therapy was associated with an altered microbiota in treated versus untreated MS patients. We found that treated patients had increases in the *Prevotella* and *Sutterella* as detected by Roche 454 and by Illumina sequencing (Fig. 2c, Table 2, Supplementary Fig. 8). Since these genera are either significantly reduced or show a trend of reduced populations in untreated patients compared with controls, it suggests that treatment with immunomodulatory therapy may normalize some of the MS-related changes in the microbiota. The genus *Sarcina* was reduced in treated versus untreated MS patients by both sequencing platforms (Fig. 2b,c). However, *Sarcina* levels were similar between untreated patients and controls, suggesting a treatment-associated effect. No significant differences in the microbiota were noted when MS patients treated with interferon therapy were compared with those treated with glatiramer acetate, although the sample sizes of these subsets were not sufficiently powered to evaluate this comparison.

To determine whether the differences we observed were related to age, gender or body mass index (BMI), we reanalysed our data using a multi-factorial model. For all MS patients (treated and untreated) compared with controls, the differences in relative abundances for the genera *Methanobrevibacter*, *Akkermansia* and *Butyricimonas* remained significant with at least one of the sequencing platforms. We also found that treatment-related differences in relative abundances for the genera *Prevotella*, *Sutterella* and *Sarcina* remained significant.

### Phylogenetic placement of 16S rRNA sequences

Validating bacterial identification is important to select the optimal bacteria to monitor in translational studies, however, partial 16S rRNA reads do not always provide sufficient information for identification to the species level. Thus, phylogenetic placement of 16S rRNA sequences was used to further identify taxa of interest and to assess the accuracy of identification using pplacer^16^ (Supplementary Figs 9 and 10). The representative sequence from the most prevalent OTU was used for each taxa of interest. The *Methanobrevibacter* sequence from our study placed most closely to reference *M. smithii* (placement likelihood (PL)=0.94), *Akkermansia* placed most closely to *A. muciniphila* (PL=1.00), and *Butyricimonas* had a greater likelihood of identification as *B. synergistica* (PL=0.83) than *B. virosa* (PL=0.50). For genera found to be different between untreated MS patients and controls, *Collinsella* placed most closely to *C. aerofaciens* (PL=0.85), *Slackia* placed closer to *S. isoflavoniconvertens* (PL=1.0 than to *S. piriformis* (PL=0.76), *Prevotella* placed closer to *P. stercorea* (PL=1.0) than to two different *P. copri* reference strains (PL=0.76 and 0.81). For taxa found to be different between untreated and treated MS patients, *Sutterella* could not be resolved between *S. stercoricanis* or *S. wadsworthensis* (PL=1.0 and 1.0, respectively) and sequences mapped to the genus *Sarcina* placed closest to *S. ventriculi* (two sequences placing near *S. ventriculi* with likelihoods of 0.66 and 0.85). In total, pplacer was able to identify the most likely genus and species from currently available reference databases.

### Gene expression in peripheral blood T cells and monocytes

We used the Nanostring immunology panel to measure the expression of 568 immune-related genes from peripheral blood derived CD4^+^ T cells and CD14^+^ monocytes from a subset of 18 MS patients and 18 controls subjects. Because *Methanobrevibacter* was significantly increased in MS patients compared with healthy subjects, and since *Methanobrevibacter* has been previously implicated as a proinflammatory microbe^17^, we selected MS patients and healthy subjects from our cohort that had the highest and lowest populations of *Methanobrevibacter*. The demographics and disease characteristics of MS patients and healthy subjects within this subset were similar to those of the larger group (Supplementary Table 1).

**Figure 3:**
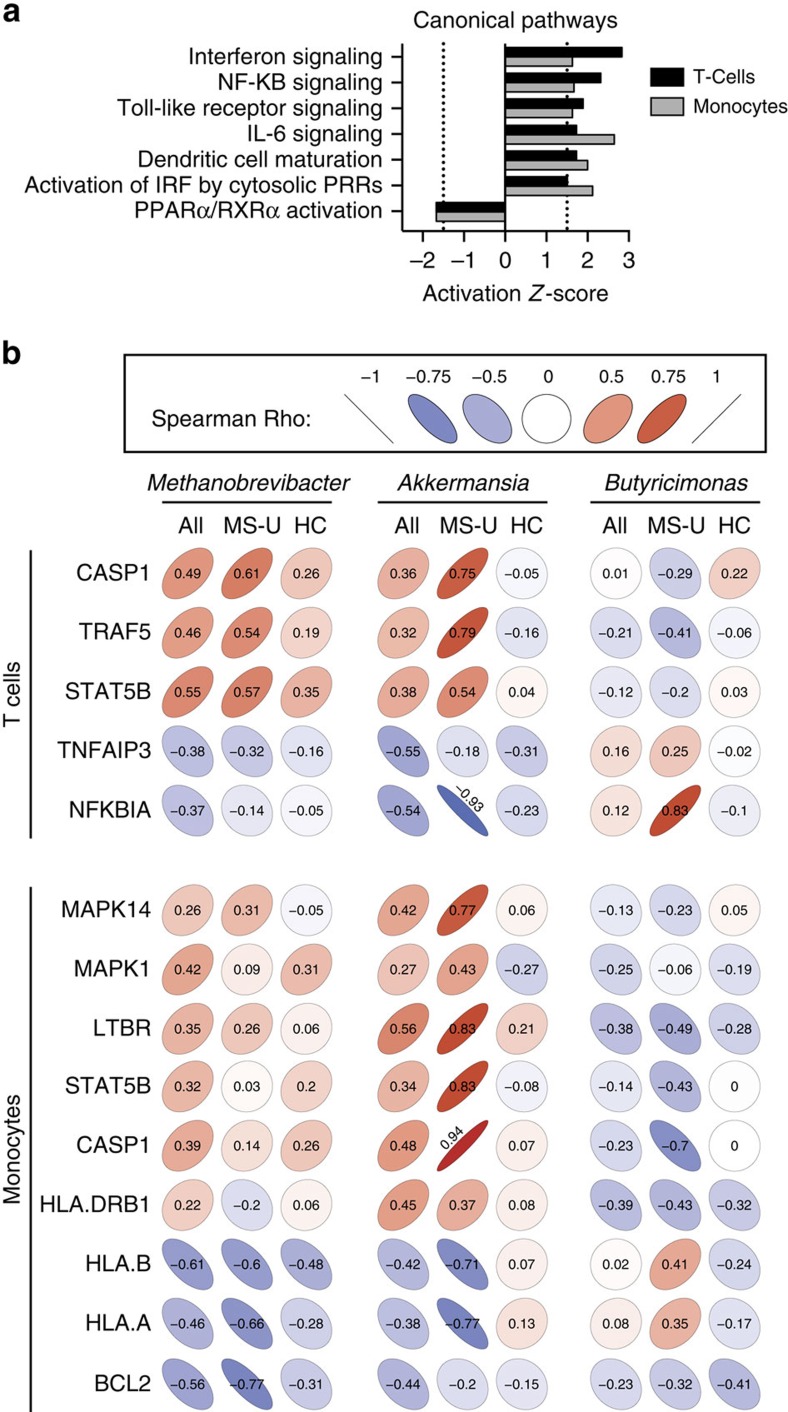
Correlations between microbiota abundances and immune gene expression. (**a**) Gene expression was measured from circulating T cells and monocytes by the Nanostring Immunology panel in MS patients (*n*=18) and healthy controls (*n*=18). (**a**) canonical pathways significantly altered in MS patients and healthy subjects with an activation *z*-score>|1.5| in both T cells (black bars) and monocytes (grey bars) identified by Ingenuity Pathway Analysis. (**b**) Altered gut microbiota abundances correlate with immune gene expression in MS patients. Spearman's correlations (*σ*) between the relative abundance of significantly altered microbes in subject groups and the relative expression of genes from identified canonical pathways significantly altered between healthy controls and untreated MS patients. Colour and slope of ellipse indicate magnitude of correlation, with *σ* value superimposed on ellipse. Subject groups were either all MS patients and controls together (All), untreated MS patients alone (MS-U) or healthy controls alone (HC).

We found unique immune transcriptional profiles in T cells and monocytes from MS patients compared with healthy controls (Supplementary Fig. 11a–c). Ingenuity pathway analysis was used to identify altered canonical pathways. Both T cells and monocytes were predicted to have activated interferon, NF-KB, Toll-like receptor and IL-6 signalling pathways, and decreased PPARα/RXRα, consistent with pathways previously correlated with MS^18^,^19^,^20^,^21^,^22^,^23^ (Fig. 3a, Supplementary Fig. 11d).

We then investigated potential associations between the MS-related microbiota and immune changes. We correlated microbial abundance with a set of significantly altered genes biologically curated from the identified canonical pathways (Fig. 3b). For T cells from all subjects, we found that both *Methanobrevibacter* and *Akkermansia* had positive correlations with CASP1, TRAF5 and STAT5B, while *Butyricimonas* had negative correlations with these genes (Fig. 3b), which are implicated in IFN signalling, IL-2 signalling, and PPAR and RXR activation. Because the correlations between microbial populations and immune expression levels could driven by the disease, we examined the correlations within untreated MS patients and within healthy controls separately. Among untreated MS patients, we again noted that *Methanobrevibacter* and *Akkermansia* had positive correlations and *Butyricimonas* had negative correlations with this set of genes, while the correlations were near zero among healthy controls alone. *Methanobrevibacter* and *Akkermansia* also had negative correlations with TNFA1P3, a known potent anti-inflammatory cytokine in autoimmune demyelination, along with NFKBIA previously known to be underexpressed in MS^20^,^21^. *Butyricimonas* had positive correlations with these two genes (Fig. 3b).

In monocytes from MS patients, *Methanobrevibacter* and *Akkermansia* had positive correlations with MAPK14, MAPK1, LTBR, STAT5B, CASP1 and HLA-DRB1, while *Butyricimonas* had negative correlations with these genes, which are implicated in dendritic cell maturation, IFN signalling and TREM signalling pathways. Among untreated MS patients, *Akkermansia* and *Butyricimonas* had positive and negative correlations, respectively, with this set of genes, whereas these effects were not observed in controls (Fig. 3b). *Methanobrevibacter* and *Akkermansia* also had negative correlations with HLA-A, HLA-B and BCL2 in untreated MS patients.

Since we observed correlations between the abundance of specific microbes and gene expression in monocytes and T cells from MS patients, we were interested in determining whether these organisms might drive an altered immunologic response in MS patients and controls. Because lipids derived from *Methanobrevibacter* are reported to have adjuvant effects^24^, we investigated *Methanobrevibacter smithii* induced proliferation and cytokine production in human peripheral blood mononuclear cells (PBMCs), but found no difference in the response to *Methanobrevibacter* in MS patients versus controls or in subjects with high *Methanobrevibacter* in the gut (Supplementary Fig. 12).

### Relationship of the gut microbiome to humoral responses

We then examined whether sera from MS patients and healthy subjects contained antibodies reactive to *Methanobrevibacter* or components of *Methanobrevibacter*. We found anti-*Methanobrevibacter* IgM, IgG and IgA antibodies (titre >1:64) as measured by enzyme-linked immunosorbent assay (ELISA) against lysates of *Methanobrevibacter* in 33% of MS patients and 28% of controls, with no differences in anti-*Methanobrevibacter* antibody titres between the two groups.

### Breath methane in MS

**Figure 4:**
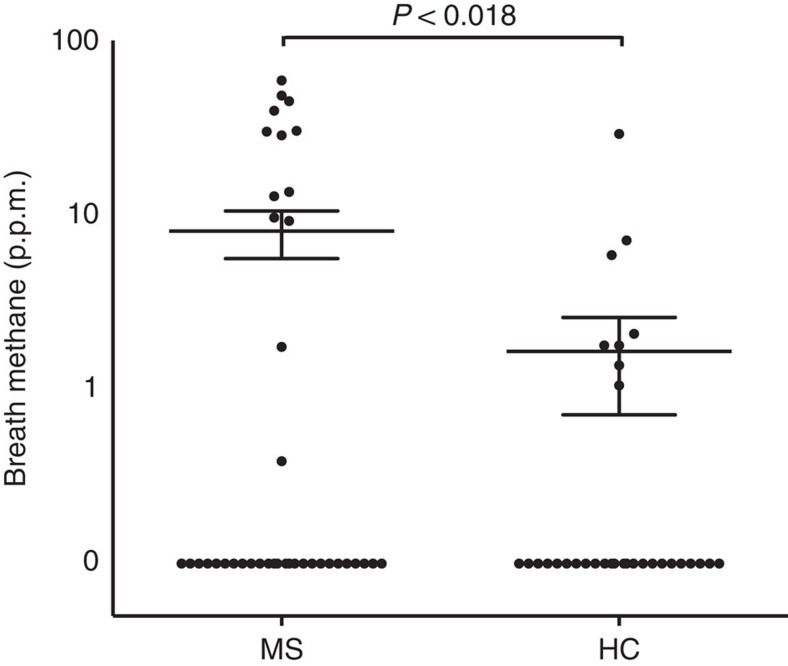
Measurement of breath methane production in MS patients (*n*=41) and controls (*n*=32). Breath methane measured in each subject is represented on the *y* axis in parts-per-million on a logarithmic scale. The mean and s.e.m. are shown by the indicated horizontal lines. 28 of 41 MS patients and 24 of 32 controls had no detectable breath methane.

Because *Methanobrevibacter* is the dominant methane-producing microbe that inhabits the human gastrointestinal tract, the likely presence of *Methanobrevibacter* in the gut can be measured indirectly by a breath test, which assesses exhaled methane by gas chromatography^25^. Detection of >1 parts-per-million of methane in the breath reflects the presence of at least 10^7^–10^8^ methanogens per gram of stool and has been shown to be driven by *Methanobrevibacter smithii*^26^. After we discovered increased *Methanobrevibacter* in the gut by 16S rRNA sequencing, we performed a methane breath test on a second cohort of 41 MS patients and 32 controls to determine whether we could verify the presence of methane-producing organisms by a simple and independent *in vivo* measurement. The second cohort was chosen to resemble our first cohort (Supplementary Table 2). We detected the presence of methane in 13/41 MS patients versus. 8/32 controls. MS patients had elevated levels of breath methane versus controls (8.07±2.46 versus 1.65±0.93 ppm, (*t*-test, *P* value<1.81 × 10^−2^), consistent with our observation of increased methanogens in the gut of MS patients (Fig. 4).

## Discussion

Host-commensal interactions have increasingly been shown to play a role in the induction of autoimmunity both in experimental animals and human diseases including inflammatory bowel disease, rheumatoid arthritis, type 1 diabetes and experimental autoimmune encephalomyelitis^8^,^27^,^28^. The origin of the autoimmune process in multiple sclerosis is still poorly understood and whether the inciting factors that trigger inflammation primarily occur in the central nervous system or in the periphery is unknown. Given that disease concordance in MS is 25% in monozygotic twins, both genetic and environmental factors likely contribute to the development of disease^29^, and the gut microbiota might be one such environmental factor.

We undertook studies to define the community structure of the faecal microbiome in MS patients using high-throughput 16S rRNA gene sequencing. We found alterations at the phylum level with increases in Euryarchaeota and Verrucomicrobia. At the genus level, more specific shifts were observed, including increases of *Methanobrevibacter* and *Akkermansia* and reduction in *Butyricimonas*. Given that discrepancies may occur due to variability in primer selection and sequencing reads, we utilized two separate sequencing methodologies and observed that the majority of taxonomic shifts were consistent in both platforms.

Several of the organisms identified in this study as being altered in MS have been demonstrated to drive inflammation or have been associated with autoimmunity. The archaeon *Methanobrevibacter* has been implicated in inflammation by its capacity to recruit inflammatory cells and activate human dendritic cells^17^ and its role in inflammatory diseases, including periodontitis, asthma and inflammatory bowel disease^24^,^30^,^31^. In addition, archaeosomes derived from *Methanobrevibacter* have potent adjuvant properties secondary to their unique lipid structure and have been used as adjuvants for vaccines^32^. *Methanobrevibacter* is distributed throughout the small bowel and colon and is tightly adherent to the mucosa via regulated expression of adhesin-like proteins, placing it in close proximity to the gut-associated lymphoid tissue^33^. Consequently, when mucosa-associated (rather than luminal) microbiota were studied in inflammatory bowel disease, *Methanobrevibacter smithii* was increased more than threefold in mucosal samples from patients with both Crohn's disease and ulcerative colitis compared to controls^30^. *Methanobrevibacter smithii* is also recovered more frequently in children with obesity, a known risk factor for the development of MS in adult life^34^. Furthermore, in a pilot study of pediatric multiple sclerosis, children colonized with *Methanobrevibacter* had a shorter time to relapse^35^.

We also found that the phylum Verrucomicrobia was increased in MS patients and was driven by the genus *Akkermansia*, which was also reported in a pilot study of 7 MS patients^36^. In contrast to our findings in MS, *Akkermansia* species have been reported to be decreased in other autoimmune diseases including psoriatic arthritis^37^. *Akkermansia* has been reported to have both regulatory and inflammatory properties, and is a mucin-degrader that converts mucin to short-chain fatty acids that may mediate the immunoregulatory effects^38^. Alternatively, *Akkermansia* has been correlated to proinflammatory pathways including upregulation of genes involved in antigen-presentation, B- and T-cell receptor signalling, and activation of complement and coagulation cascades^39^. These proinflammatory features may be related to its ability to degrade mucus, leading to breakdown of the gut barrier and increased exposure of resident immune cells to microbial antigens^40^.

We found lower abundances of *Butyricimonas,* a butyrate-producing genus, in MS patients. Butyrate is a short-chain fatty acid produced by microbes that induce colonic regulatory T cells^41^. Reductions in colonic butyrate can disrupt barrier function and promote inflammation. Similar to our findings, reductions in butyrate producers have been noted in numerous autoimmune and inflammatory diseases including inflammatory bowel disease, rheumatoid arthritis and type 1 diabetes^5^,^42^,^43^.

We investigated untreated MS patients, to examine the microbiota independent of MS disease-modifying therapy. We found that the genera that were altered in the entire MS cohort (*Methanobrevibacter*, *Akkermansia* and *Butyricimonas*) were also altered in the untreated population, suggesting that these effects are not specifically correlated with therapy. Furthermore, within the untreated MS subset, we observed reductions in genera belonging to the family Coriobacteriaceae, including *Collinsella* and *Slackia*. Reductions in Coriobacteriaceae have been reported in relatives of patients with inflammatory bowel disease^44^.

Patients on disease-modifying therapy had increased abundances of the genera *Prevotella* compared with untreated patients. Although *Prevotella* has been reported to be increased in rheumatoid arthritis and inflammatory bowel disease^5^, *Prevotella* has been previously correlated to the intake of high-fibre diets, whose primary substrate, fibre, can drive the generation of the immunoregulatory metabolite butyrate^45^. We found that *Prevotella* was low in untreated MS, and that treatment with disease-modifying therapy was associated with increased relative abundance of *Prevotella.* In a smaller cohort of 20 MS patients compared with 40 controls in Japan, the authors detected a decrease in *Prevotella*^11^ in MS. Given this consistent finding, future studies investigating the role of *Prevotella* in MS are warranted.

We also observed increases in the genus *Sutterella* and decreases in *Sarcina* in MS patients on therapy. *Sutterella* was found to be increased in healthy controls compared to patients with new-onset Crohn's disease^46^. *Sarcina* species are reported to be increased in the gut microbiota of autistic patients^47^. Since immunomodulatory treatment in MS was associated with increases in relative abundances of *Prevotella* and *Sutterella* and decreases in *Sarcina*, it is conceivable that treatment may act to normalize a proinflammatory microbiota.

Our finding that some of MS patients have elevated exhaled methane, a surrogate for levels of *Methanobrevibacter* in the gut, is consistent with our 16S rRNA sequencing results; it would be interesting to use this rapid, *in vivo* test to investigate *Methanobrevibacter* in larger populations of MS patients. While we did not measure stool abundance of *Methanobrevibacter* in this second cohort, previous studies have shown that the amount of breath methane strongly correlates with the quantity of *M. smithii* in the stool^26^. In other anatomical sites, the presence of particular microbial species can be identified based on their metabolic activity, as is routinely done for diagnosing *Helicobacter pylori* presence with a positive gastric urease test. Future studies employing simultaneous collection of breath, faecal and blood samples in MS patients will address the potential role of breath methane as a biomarker in MS.

The gut microbiota are known to modulate host immune gene expression either by direct contact with cell wall components, or by secretion of factors that can signal through host receptors or through epigenetic modifications that may alter methylation or acetylation of transcriptional promoters^48^. Microbial colonization by a single organism or by groups of organisms into the gut of germ-free mice can modulate the expression of innate and adaptive immune genes as early as 4 days after microbial inoculation in varying cellular compartments^48^. In a study of inflammatory bowel disease in humans, associations between microbes and host gene expression were found in innate and adaptive immune pathways^49^.

We examined relationships between microbial abundance and immune genes implicated in MS pathogenesis. Consistent with the inflammatory properties of *Methanobrevibacter* and *Akkermansia*^32^,^33^,^34^,^36^,^45^,^46^, we found positive correlations with these organisms and gene expression in T cells and monocytes involved in key pathways previously implicated in MS pathogenesis, including increased expression of the MAPK family in monocytes (MAPK1 and MAPK14), genes directly involved in both the initiation phase of innate immunity and activating adaptive immunity^50^. In T cells, *Methanobrevibacter* and *Akkermansia* positively correlated with TRAF5, a known regulator of T-cell activation and known to be overexpressed in MS, as well as STAT5B, whose expression is indispensible for the encephalitogenicity of autoreactive CD4^+^ T cells in EAE^50^,^51^. *Methanobrevibacter* or *Akkermansia* negatively correlated with TNFAIP3, previously shown to have reduced expression in studies of the MS transcriptome^21^,^50^. In the case of *Akkermansia*, these relationships were even stronger among untreated MS patients alone. *Butyricimonas* had negative correlations with genes known to be increased in MS among T cells and monocytes, suggesting that reduction in *Butyricimonas* is associated with increased proinflammatory gene expression, however, directionality cannot be determined from this study. These correlations were observed among all subjects and in untreated MS patients, but not in controls alone, suggesting that the relationship between microbial abundance and gene expression was MS-specific, but not solely driven by differences between controls and MS patients. Although we could detect correlation, we cannot determine the causal direction or conclude whether the microbes drive immunological changes, or whether the disease or altered immunity drives changes in the microbiota.

While our investigation of the gut microbiota in MS provides initial insights into understanding the potential role for the microbiome in this disease, our study has certain limitations. First, we cannot assign a direct cause to the associations we describe in the gut microbiome and the MS immunophenotype. The altered microbes may play a role in disease-related immunological changes, or MS-related changes in physiology may drive microbial alterations. *Methanobrevibacter*, for example, is recovered more frequently from individuals with constipation-variant irritable bowel disease^52^; while we excluded patients with irritable bowel disease from our study, it is possible, for example, that subtle changes in gut motility or in the enteric nervous system in MS patients or co-existing constipation may produce conditions favourable for the growth of this microbe. Second, our findings could be influenced by cohort-specific confounders. To minimize the effects of confounders, we used strict exclusion criteria eliminating the potential influence of antibiotics, pregnancy or other autoimmune or gastrointestinal conditions. Medications taken before the window of our study—such as prior courses of steroids—may also influence gut microbial populations. Furthermore, MS patients often consume alternative diets that might favour the growth of particular microbial niches^53^. Although we did not find any large differences in dietary intake in our cohort, more sensitive assays may reveal dietary variation that may be responsible for the observed changes in the gut microbiome in our disease population. Although the methodology used to collect stool samples was relatively uniform, variability in time of sample collection and interval of previous dietary intake may have also contributed to changes in microbes recovered. While age, gender and BMI have been previously shown to drive changes in the microbiome, our multi-factorial model suggests that these factors did not confound the observed microbial differences between the groups. Although we did not find a relationship between changes in the gut microbiome and clinical parameters such as disease duration or disability, most of our patients had low levels of disability (average EDSS 1.2). Future studies with larger cohorts and longitudinal collection of samples will be required to investigate these clinical associations, including subjects with progressive forms of the disease. Third, we analysed the microbiota using primers targeting the 16S rRNA gene. While this is useful for providing taxonomic information, shotgun metagenomic, metatranscriptomic and metabolomic profiling of faecal samples may reveal changes in the abundance of microbial genes, their expression or the presence of microbial metabolites that would complement phylogenic and taxonomic data. Finally, our study is limited by the fact that we collected samples after disease onset. It is possible that critical changes in the gut microbiome in MS may occur in early or preclinical stages of disease. For example, in type 1 diabetes, changes in the gut microbiome were apparent before the onset of illness in high-risk individuals with the concomitant appearance of anti-islet antibodies^54^. Investigation of the microbiota in paediatric or early-onset MS may provide further evidence of associations between the composition of the gut microbiome and MS pathogenesis.

In summary, we have found alterations of the human gut microbiome in MS that correlate with changes in the immune transcriptome and treatment. It is possible that treatment strategies of MS in the future may include therapeutic interventions designed to affect the microbiome such as probiotics, faecal transplantation and delivery of constituents of organisms isolated from the microbiome^10^, although more work is required. In addition, characterization of the gut microbiome in MS may provide biomarkers for assessing disease activity and could theoretically be an avenue to prevent MS in young at-risk populations.

## Methods

### Study population

Relapsing–remitting MS patients were recruited from the Partners MS Center at Brigham and Women's Hospital and healthy subjects from the Brigham and Women's Hospital PhenoGenetic project (http://dejager_lab.bwh.harvard.edu/?page_id=2317). Untreated patients were treatment naive or with no steroid treatment in the previous month, no beta-interferon/glatiramer acetate treatment in the previous 3 months, and no other treatments over the prior 6 months. None of the MS patients had an active relapse at the time of sampling. Patients with a history of using other immunosuppressive medications including teriflunomide, cyclophosphamide, mitoxantrone, rituximab, intravenous immunoglobulin, daclizumab, basiliximab, azathioprine, methotrexate or mycophenolate mofetil were excluded. Treated patients in the cohort were those who had received beta-interferon or glatiramer acetate for at least 6 months. Exclusion criteria for both MS subjects and healthy control subjects were as follows: no antibiotic use in the prior 6 months; no probiotic use; corticosteroids; history of gastroenteritis; or travel outside of the country in the prior month. No history of irritable bowel syndrome, bowel surgery, inflammatory bowel disease or other autoimmune disease. Pregnancy was also an exclusion criteria. A dietary survey was administered to all subjects before collection of samples (Supplementary Table 3). The protocol was approved by the Partners Human Research Committee and informed consent was obtained from all subjects.

### Sample collection

Stool samples were obtained from patients by providing them with stool collection containers. We used a consistent methodology for processing and storage of all samples. Subjects collected a single-sample produced at any time of day with no specific dietary restrictions. Collection containers were then placed in boxes with provided ice packs for immediate shipment to our laboratory via overnight delivery at a maintained temperature of 0 °C. On receipt of samples, they were frozen at −80 °C until DNA extraction^55^. Samples were only subjected to a single free-thaw cycle.

### Preparation of DNA and sequencing protocols

For Roche 454 pyrosequencing, DNA was extracted using the PowerSoil DNA Isolation kit (MO BIO Laboratories, Carlsbad, CA, USA) with the Human Microbiome Project modifications to the manufacturer's protocol^56^. DNA quality and yield were evaluated via agarose gel and Qubit fluorometer (Life Technologies Corporation, Carlsbad, CA, USA). The 16S rRNA gene libraries were generated by the Center for Metagenomics and Microbiome Research at Baylor College of Medicine, using the V3–V5 (357F/926R) primer in accordance with standard Human Microbiome Project protocols^55^. The 16S rRNA libraries were sequenced by the Human Genome Sequencing Center at Baylor College of Medicine using a Roche 454 GS FLX+ instrument (Roche, Indianapolis, IN) operated with Titanium chemistry.

For Illumina MiSeq 16S rRNA sequencing, methods were adapted from the protocol developed for the NIH-Human Microbiome Project^55^. Briefly, bacterial genomic DNA was extracted using the MO BIO PowerSoil DNA Isolation Kit (MO BIO Laboratories). The 16S rRNA V4 region was amplified by PCR and sequenced on the MiSeq platform (Illumina) using the 2 × 150 bp paired-end protocol. The primers used for amplification contain adaptors for MiSeq sequencing and dual-index barcodes so that the PCR products may be pooled and sequenced directly^57^. Sequencing was performed at the Human Genome Sequencing Center at Baylor College of Medicine.

### 16S rRNA sequencing data preprocessing

Sequencing of the 105 human stool samples on the Roche 454 GS FLX+ instrument generated 1,426,326 total raw reads. Raw reads were processed using the mothur software package (v.1.34.2)14 (ref. 15), which performs demultiplexing and denoising, quality filtering, alignment against the ARB Silva reference database of 16S rRNA gene sequences, and clustering into OTUs (at 97% identity; Supplementary Fig. 1). In total, 4317 OTUs were generated. After filtering OTUs that failed to have a mean number of reads per sample ≥1 in at least one cohort, 426 OTUs were available for further analysis.

Sequencing of the samples on the Illumina MiSeq instrument generated 11,498,168 total forward raw reads. Preprocessing was performed using the mothur software package. The mothur MiSeq SOP was followed, except we used a custom Python script to perform base quality trimming (sliding window size of 50 nt with average quality score >35); this modification was employed to improve the quality of sequencing reads used for OTU clustering, since the standard mothur SOP assumes longer sequencing reads than generated by our MiSeq protocol. 10,620 OTUs were generated, with 1,191 OTUs available for further analysis after filtering using the same procedure as described for the Roche 454 data (Supplementary Fig 1).

### 16S rRNA sequencing data analysis

A measure of alpha diversity, the Shannon entropy^58^ was calculated for the samples, and the nonparametric Wilcoxon rank-sum test^59^ was applied for hypothesis testing. To visualize differences in overall microbial community structure, the unweighted and weighted UniFrac measures were calculated between all pairs of samples, and Principal Coordinates Analysis plots were generated using custom R scripts^60^. Analysis of molecular variance was used for statistical hypothesis testing of differences in overall microbial community structure between cohorts as assessed with the UniFrac measures^61^.

To statistically test for differences in the relative abundances of microbial taxa (phyla or genera) the DESeq2 software package was employed^62^,^63^. To control for covariates (gender, age and BMI) of interest, we used the multi-factorial model in DESeq2. The DESeq2 multi-factorial model requires categorical values if >1 covariate is included, so we discretized age and BMI values. For BMI, we used standard World Health Organization (WHO) categories of normal, overweight, and obese. We reclassified one subject with a BMI of 18.1 as normal, as this is at the borderline of the WHO underweight classification (<18.5) and would have resulted in only one subject in the underweight category. For age, we divided the range of study subjects' ages (27 to 63 years) into four categories, with each category spanning a 9-year increment. Eight subjects were missing the clinical data. These subjects were excluded from analyses controlling for covariates.

For all statistical testing for 16S rRNA data analysis, *P* values were adjusted for multiple hypothesis testing using the method of Benjamini and Hochberg^64^.

To more accurately identify the microorganisms present in samples and their phylogenetic relationships to known species, the pplacer software package was used to perform phylogenetic placement^16^. Pplacer uses a likelihood-based methodology to place short sequencing reads of 16S rRNA amplicons on a reference tree, and also generates taxonomic classifications of the short sequencing reads using a least common ancestor-based algorithm. The reference tree required for phylogenetic placement was generated using full-length or near full-length (>1,200 nt) 16S rRNA sequences of type strains from the Ribosomal Database Project (RDP)^65^. 9,563 16S rRNA sequences were downloaded from RDP-11-1, representing 8,719 type strains with 375 sequences from Archaea and 9,188 sequences from Bacteria strains. Filtering was performed using custom Python scripts with the following criteria: (1) only one sequence per species (if the species had more than one sequence, then the longest sequence was chosen); (2) non-environmental species (using key words); (3) no sequences with unclear taxonomic lineage information; (4) no redundant sequences. 7,890 16S rRNA sequences were retained for constructing the phylogenetic tree. Multiple sequence alignment was performed using Muscle^66^. Per the pplacer manual (http://matsen.github.io/pplacer/generated_rst/pplacer.html), the 16S rRNA reference tree was constructed using FastTree with parameter ‘-nt –gtr' (ref. 67). FastTree infers phylogenetic trees using an approximate maximum likelihood-based approach, and generates local support values to estimate the reliability of each split in the tree by using the Shimodaira–Hasegawa test on the three alternate topologies (NNIs) around that split, counting the fraction of 1,000 resamples that support a split over the two potential NNIs around that node. Local support values of >0.95 are considered to strongly support splits, and those >0.70 are considered to moderately support splits. To generate the phylogenetic placement figures in the main manuscript (Supplementary Figs 9 and 10), representative sequences for each species within the genus of interest were chosen (the most abundant unique sequence assigned to that species by pplacer).

### Methane breath testing

A separate cohort of subjects was utilized for methane breath testing. The inclusion and exclusion criteria used to recruit patients was identical to that described above. Breath samples were collected from subjects after an overnight fast. All subjects brushed their teeth following the 8-hour fast then produced end-expiratory breath samples into provided collection tubes. Collection tubes were sent to Commonwealth Labs (Salem, MA) where gas chromatography was performed to assess the presence of exhaled methane as described previously^26^.

### Immunologic assays

The *Methanobrevibacter smithii* type strain (ATCC 35061) was cultured at the University of Massachusetts using methods previously described^68^. Assesment of antibodies against *M. Smithii* was performed by ELISA. Lysates of *Methanobrevibacter* were prepared from sonicates of *Methanobrevibacter* cultures. PBMCs were isolated as previously described^69^. PBMCs were stimulated with *Methanobrevibacter smithii*, tetanus toxoid or anti-CD3/CD28 in 15 healthy donors and 14 MS patients. *Methanobrevibacter smithii* was cultured in a ratio of 1 bacteria to 1, 10, 100 or 1,000 PBMCs. Thymidine incorporation was measured 4 days following stimulation. Cytokine production was measured by Luminex assay (Miltenyi Biotec). Monocytes and T cells were sorted from PBMCs from 20 MS patients and 20 healthy controls (see below) using a Miltenyi Biotec (Alburn, CA) selection kit. Total RNA was extracted using Norgen RNA purification kits (Norgen Biotek Corp., norgenbiotek.com). NanoString expression of immune genes was detected by NanoString array (nCounter, Gene expression code set, Human Immunology kit) as previously described^69^.

### Statistical analyses of gene expression and microbiome associations

T cells and monocytes were sorted from PBMCs from 20 MS patients and 20 healthy subjects. These participants were chosen from the larger cohort by selecting 10 MS patients with the highest *Methanobrevibacter* relative abundance, 10 MS patients with the lowest *Methanobrevibacter* relative abundance, 10 healthy subjects with the highest *Methanobrevibacter* relative abundance and 10 healthy subjects with the lowest *Methanobrevibacter* relative abundance. Of these samples, T cells and monocytes from two MS patients and two healthy subjects either had low viability of low recovery of RNA thus these samples were excluded from further analysis. Count data were normalized using the nSolver Analysis software (Nanostring), significant differences were detected by *t*-test and *P* values were adjusted for multiple comparisons using the Benjamini–Hochberg correction to assess significance at the 0.05 level.

Pathway annotations were conducted using the Ingenuity Pathway Analysis (Qiagen) and canonical pathways that had an activation score >1.5 or <−1.5 in either T cells or monocytes were reported. Spearman's correlations between microbial abundance and immune gene expression was calculated in the R statistical framework^70^ using the cor.test in the stats package and plotted using the ellipse package^71^.

### Data availability

The high-throughput sequence data have been deposited in the National Center for Biotechnology Information (NCBI) BioProject database with project number PRJNA321051. The data from the 16S rRNA sequencing on the MiSeq and 454 platforms have been deposited in the NCBI Sequence Read Archive, and linked to the mentioned BioProject number. T-cell and monocyte gene expression data obtained using the Nanostring immunology panel have been deposited in the NCBI Gene Expression Omnibus (GEO) database under accession number GSE81279. The authors declare that all other data supporting the findings of this study are available within the article and its Supplementary Information files, or from the corresponding author on request.

## Additional information

**How to cite this article:** Jangi, S. *et al.* Alterations of the human gut microbiome in multiple sclerosis. *Nat. Commun.* 7:12015 doi: 10.1038/ncomms12015 (2016).

## Supplementary Material

Supplementary Information Supplementary Figures 1-12 and Supplementary Tables 1-3

## Figures and Tables

**Table 1:** Demographics of study population.

	**Healthy**	**Multiple sclerosis**
	***N*****=43**	***N*****=60**
Age	42.2±9.61	49.7±8.50
Male (%)	6 (14%)	19 (32%)
Female (%)	37 (86%)	41 (68%)
Body mass index	26.4±6.3	27.2±4.7
Caucasian	43	58
Black	0	2
Hispanic	0	1
Disease Duration	NA	12.8±8.3
EDSS Score	NA	1.2 ±1.0
Untreated	NA	28
Beta-interferon	NA	18
Glatiramer acetate	NA	14

NA, not applicable

**Table 2:** Multiple sclerosis and MS-treatment-associated taxa.

	**Relative abundances**								**BH corrected** *P* **values**					
	**454**				**Illumina**				**HC vs MS**		**HC versus Un**		**Tr vs Untreated**	
	**HC**	**All MS**	**Untr MS**	**Tr MS**	**HC**	**All MS**	**Untr MS**	**Tr MS**	**454**	**MiSeq**	**454**	**MiSeq**	**454**	**MiSeq**
*Phyla*														
*Euryarcheota*	8.65 × 10^−5^	5.48 × 10^−4^	3.36 × 10^−4^	3.12 × 10^−4^	3.66 × 10^−3^	1.38 × 10^−2^	1.17 × 10^−2^	8.53 × 10^−3^	**1.38** × **10**^−**2**^	**2.74** × **10**^−**2**^	1.88 × 10^−2^	n.s.	n.s.	n.s.
*Verrucomicrobia*	8.06 × 10^−3^	1.65 × 10^−2^	2.25 × 10^−2^	5.80 × 10^−3^	5.30 × 10^−2^	1.07 × 10^−1^	1.39 × 10^−1^	6.18 × 10^−2^	**1.77** × **10**^−**5**^	**3.66** × **10**^−**3**^	**8.91** × **10**^−**4**^	**1.41** × **10**^−**2**^	n.s.	n.s.
														
*Genera*														
*Methanobrevibacter*	8.65 × 10^−5^	5.48 × 10^−4^	3.37 × 10^−4^	3.12 × 10^−4^	3.64 × 10^−3^	1.37 × 10^−2^	1.16 × 10^−2^	8.40 × 10^−3^	**7.80** × **10**^−**3**^	**2.30** × **10**^−**3**^	1.28 × 10^−2^	n.s.	n.s.	n.s.
*Akkermansia*	8.06 × 10^−3^	1.65 × 10^−2^	2.25 × 10^−2^	5.80 × 10^−3^	5.29 × 10^−2^	1.07 × 10^−1^	1.39 × 10^−1^	6.18 × 10^−2^	**9.00** × **10**^−**4**^	**3.45** × **10**^−**2**^	**3.20** × **10**^−**3**^	**3.36** × **10**^−**2**^	n.s.	n.s.
*Butyricimonas*	1.16 × 10^−3^	3.75 × 10^−4^	2.80 × 10^−4^	4.11 × 10^−4^	1.37 × 10^−3^	5.81 × 10^−4^	4.26 × 10^−4^	7.21 × 10^−4^	**3.50** × **10**^−**3**^	**9.50** × **10**^−**3**^	**1.31** × **10**^−**2**^	**6.70** × **10**^−**3**^	n.s.	n.s.
*Paraprevotella*	3.57 × 10^−4^	2.18 × 10^−5^	0	3.84 × 10^−5^	3.47 × 10^−3^	4.65 × 10^−4^	3.44 × 10^−5^	7.73 × 10^−4^	**3.45** × **10**^−**2**^	**8.70** × **10**^−**3**^	**9.52** × **10**^−**2**^	**1.50** × **10**^−**3**^	n.s.	n.s.
*Haemophilus*	3.95 × 10^−3^	3.32 × 10^−3^	5.25 × 10^−3^	2.68 × 10^−3^	4.61 × 10^−3^	4.79 × 10^−3^	7.75 × 10^−3^	3.83 × 10^−3^	**3.50** × **10**^−**3**^	**2.57** × **10**^−**2**^	**2.10** × **10**^−**3**^	**9.60** × **10**^−**3**^	n.s.	6.00 × 10^−4^
*Slackia*	1.99 × 10^−3^	5.50 × 10^−4^	5.00 × 10^−4^	5.56 × 10^−4^	1.62 × 10^−4^	4.77 × 10^−5^	6.17 × 10^−5^	4.40 × 10^−5^	**3.60** × **10**^−**3**^	**5.21** × **10**^−**2**^	**2.5** × **10**^−**5**^	**1.35** × **10**^−**2**^	6.55 × 10^−2^	n.s.
*Collinsella*	5.54 × 10^−3^	4.90 × 10^−3^	3.61 × 10^3^	6.10 × 10^3^	2.27 × 10^3^	1.52 × 10^−3^	1.16 × 10^−3^	1.89 × 10^−3^	n.s.	n.s.	**8.19** × **10**^−**2**^	**4.30** × **10**^−**3**^	n.s.	9.20 × 10^−3^
*Megasphaera*	1.29 × 10^−3^	8.18 × 10^−3^	2.05 × 10^−2^	1.57 × 10^−3^	1.46 × 10^−3^	8.67 × 10^−3^	2.06 × 10^−2^	2.45 × 10^−3^	9.50 × 10^−3^	n.s.	**1.00** × **10**^−**4**^	**3.84** × **10**^−**2**^	**1.90** × **10**^−**7**^	**5.50** × **10**^−**5**^
*Cloacibacillus*	4.26 × 10^−5^	2.64 × 10^−4^	5.93 × 10^−4^	4.41 × 10^−5^	0	1.06 × 10^−5^	2.34 × 10^−5^	1.50 × 10^−6^	3.20 × 10^−2^	n.s.	**4.98** × **10**^−**2**^	**9.20** × **10**^−**3**^	**6.92** × **10**^−**2**^	**3.45** × **10**^−**2**^
Veillonellaceae_unc	8 × 10^−3^	6.12 × 10^−4^	9.21 × 10^−4^	3.36 × 10^−4^	1.14 × 10^−3^	1.91 × 10^−4^	3.56 × 10^−4^	1.12 × 10^−4^	n.s.	n.s.	**8.51** × **10**^−**2**^	**2.38** × **10**^−**2**^	**5.17** × **10**^−**2**^	**3.45** × **10**^−**2**^
*Prevotella*	1.92 × 10^−2^	7.90 × 10^−3^	1.23 × 10^−3^	1.25 × 10^−2^	2.76 × 10^−2^	3.06 × 10^−3^	1.23 × 10^−3^	1.32 × 10^−2^	n.s.	n.s.	**7.27** × **10**^−**2**^	**2.80** × **10**^−**7**^	**9.02** × **10**^−**2**^	**2.80** × **10**^−**5**^
*Sutterella*	6.40 × 10^−3^	6.49 × 10^−3^	1.16 × 10^−4^	8.49 × 10^−4^	1.11 × 10^−4^	1.00 × 10^−4^	1.54 × 10^−5^	1.80 × 10^−4^	n.s.	n.s.	**7.64** × **10**^−**2**^	**6.80** × **10**^−**2**^	**4.20** × **10**^−**3**^	**8.70** × **10**^−**3**^
Clostridia_unc	5.05 × 10^−5^	1.77 × 10^−4^	3.17 × 10^−4^	5.25 × 10^−5^	7.51 × 10^−2^	8.51 × 10^−2^	8.51 × 10^−2^	9.07 × 10^−2^	8.51 × 10^−2^	n.s.	8.00 × 10^−3^	n.s.	**1.92** × **10**^−**2**^	**8.60** × **10**^−**3**^
*Sarcina*	2.02 × 10^−2^	1.20 × 10^−2^	2.04 × 10^−2^	5.45 × 10^−3^	1.45 × 10^−2^	8.58 × 10^−3^	1.51 × 10^−2^	4.11 × 10^−3^	6.92 × 10^−2^	n.s.	n.s.	n.s.	**9.00** × **10**^−**4**^	**2.52** × **10**^−**2**^
Mollicutes_unc	2.49 × 10^−2^	1.05 × 10^−2^	2.02 × 10^−3^	2.02 × 10^−3^	3.61 × 10^−3^	1.08 × 10^−3^	7.81 × 10^−4^	1.36 × 10^−3^	8.30 × 10^−3^	n.s.	3.00 × 10^−7^	n.s.	n.s.	n.s.
Prevotellaceae_unc	4.99 × 10^−3^	1.37 × 10^−4^	7.31 × 10^−6^	1.07 × 10^−5^	2.15 × 10^−3^	4.22 × 10^−4^	1.40 × 10^−4^	2.37 × 10^−4^	3.00 × 10^−10^	n.s.	4.40 × 10^−5^	n.s.	n.s.	n.s.
*Holdemania*	1.72 × 10^−3^	3.80 × 10^−3^	3.69 × 10^−3^	3.08 × 10^−3^	4.26 × 10^−4^	4.85 × 10^−4^	4.03 × 10^−4^	5.51 × 10^−4^	4.20 × 10^−3^	n.s.	4.02 × 10^−2^	n.s.	n.s.	n.s.
*Desulfovibrio*	9.48 × 10^−5^	1.63 × 10^−4^	3.75 × 10^−4^	3.95 × 10^−5^	1.16 × 10^−3^	9.31 × 10^−4^	1.65 × 10^−3^	4.65 × 10^−4^	n.s.	n.s.	1.58 × 10^−2^	n.s.	1.80 × 10^−3^	n.s.
Peptococcaceae_unc	8.14 × 10^−5^	1.74 × 10^−4^	3.85 × 10^−4^	5.26 × 10^−5^	2.01 × 10^−4^	2.72 × 10^−4^	2.56 × 10^−4^	2.79 × 10^−4^	n.s.	n.s	4.03 × 10^−2^	n.s.	3.30 × 10^−2^	n.s.
*Barnesiella*	4.76 × 10^−3^	2.00 × 10^−3^	1.35 × 10^−3^	1.41 × 10^−3^	1.18 × 10^−2^	6.55 × 10^−3^	8.35 × 10^−3^	5.43 × 10^−3^	n.s.	1.00 × 10^−4^	1.60 × 10^−3^	n.s.	n.s.	n.s.
Acidaminococcaceae_unc	3.45 × 10^−3^	8.43 × 10^−4^	1.46 × 10^−4^	5.01 × 10^−4^	5.70 × 10^−4^	5.82 × 10^−5^	4.14 × 10^−5^	8.51 × 10^−5^	n.s.	2.57 × 10^−2^	3.15 × 10^−2^	n.s.	n.s.	n.s.
*Megamonas*	8.54 × 10^−5^	6.34 × 10^−5^	0	1.25 × 10^−4^	1.20 × 10^−4^	2.79 × 10^−4^	2.66 × 10^−6^	5.47 × 10^−4^	n.s.	1.80 × 10^−5^	n.s.	1.54 × 10^−2^	n.s.	n.s.
*Guggenheimella*	**—**	**—**	**—**	**—**	3.17 × 10^−3^	1.62 × 10^−3^	1.94 × 10^−5^	3.16 × 10^−3^	**—**	3.60 × 10^−5^	**—**	9.50 × 10^−3^	**—**	n.s.
Thermoplasmatales_unc	**—**	**—**	**—**	**—**	1.82 × 10^−6^	2.08 × 10^−5^	2.63 × 10^−5^	1.45 × 10^−6^	**—**	3.45 × 10^−2^	**—**	1.23 × 10^−2^	**—**	1.62 × 10^−2^
*Buttiauxella*	7.77 × 10^−5^	2.45 × 10^−4^	2.01 × 10^−4^	2.77 × 10^−4^	3.32 × 10^−3^	1.32 × 10^−3^	1.41 × 10^−4^	2.49 × 10^−3^	n.s.	n.s.	n.s.	1.00 × 10^−3^	n.s.	1.90 × 10^−3^
*Sporanaerobacter*	**—**	**—**	**—**	**—**	8.78 × 10^−4^	6.45 × 10^−4^	1.43 × 10^−4^	1.04 × 10^−3^	**—**	n.s.	**—**	4.20 × 10^−4^	**—**	1.00 × 10^−4^

HC, healthy controls; MS, multiple sclerosis; unc, unclassified; Untr, untreated MS patients; Tr, treated MS patients.

The table lists taxa that differ between HC and MS patients, between HC and Untr and between untreated and Tr, detected on two different sequencing platforms, 454 and MiSeq. Significant differences were tested by DESeq, and *P* values were adjusted by the Benjamini–Hochberg method. Relative abundances are listed. Values that are significant (*P*<0.05) on both platforms are in bold. Taxa are listed at the genus level or at their lowest possible level of classification, followed by unc. Genera that were not detected on a given platform are indicated with a (**—**). Non-significant *P* values are listed as n.s.
