# Supplementary material for: Alterations of the human gut microbiome in multiple sclerosis
Source: Nat Commun. 2016 Jun 28;7:12015. doi: 10.1038/ncomms12015 (PMC4931233; doi:10.1038/ncomms12015)
Supplement: Supplementary Information — Supplementary Figures 1-12 and Supplementary Tables 1-3 [file ncomms12015-s1.pdf]

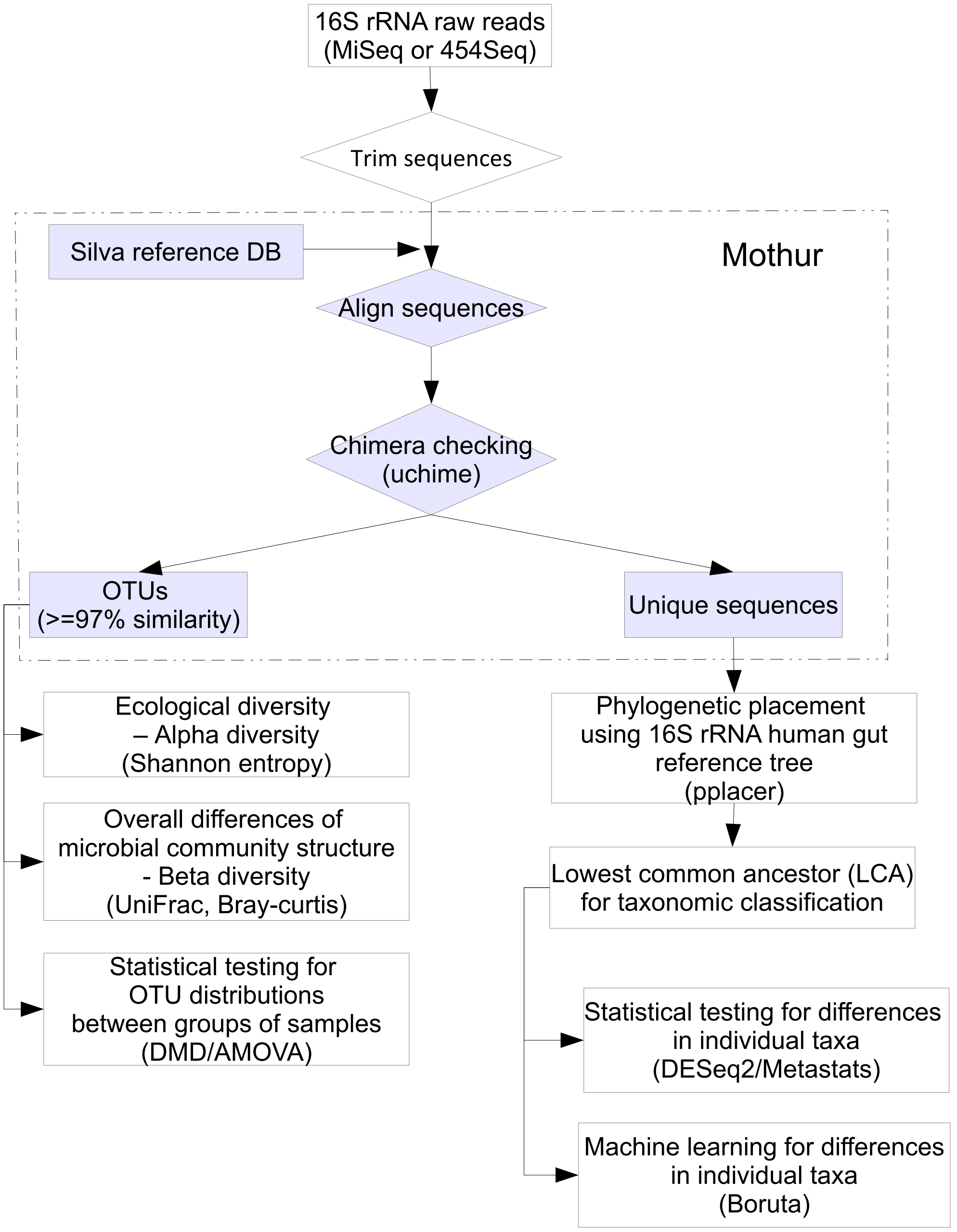

Supplementary Figure 1: Bioinformatics pipeline. Sequencing of samples was performed on both the Roche 454 GS FLX+ instrument and the Illumina MiSeq instrument. Raw reads from 454 sequencing and MiSeq were processed using the Mothur software package and clustered into Operational Taxonomic Units (OTUs) at 97% identity and then used to determine measures of ecologic diversity and community structure. Unique sequences were utilized for phylogenetic placement and subsequent taxonomic classification and to find differences in taxa between MS patients and controls.

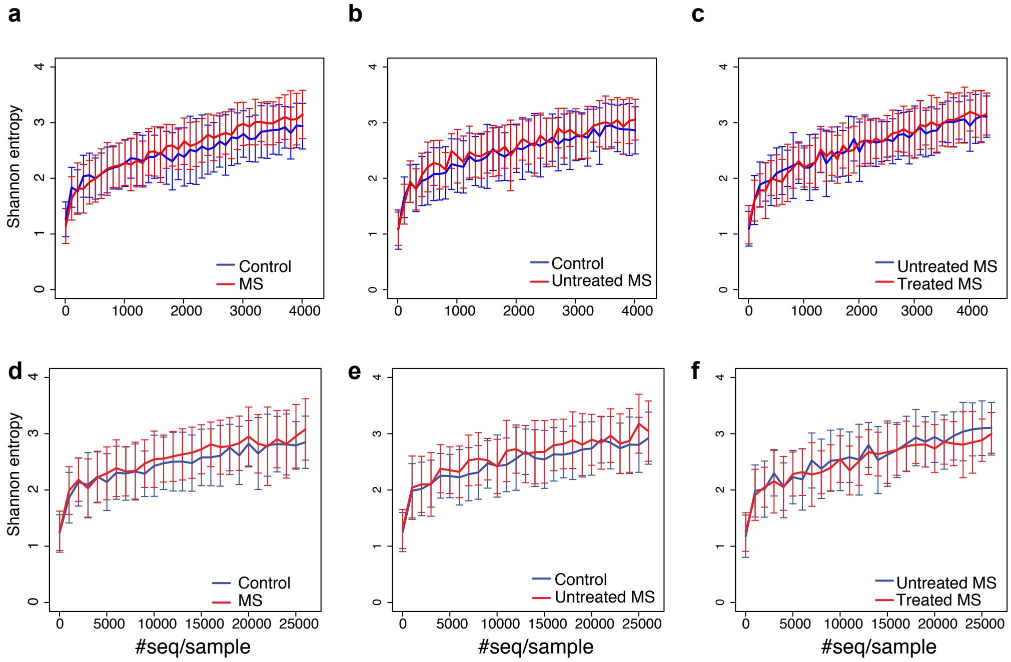

Supplementary Figure 2: Alpha-diversity of gut microbiome. Rarefaction curves were calculated at multiple sequence depths on either the 454 **(a-c)** or MiSeq **(d-f)** platform for Shannon entropy to compare differences in alpha-diversity between **a,d**) control (n = 43) and MS patients (n = 60), **b,e**) control (n = 43) and untreated MS patients (n = 28), and **c,f**) untreated (n = 28) and treated (n = 32) MS patients. Graphs depict average +/- standard error.

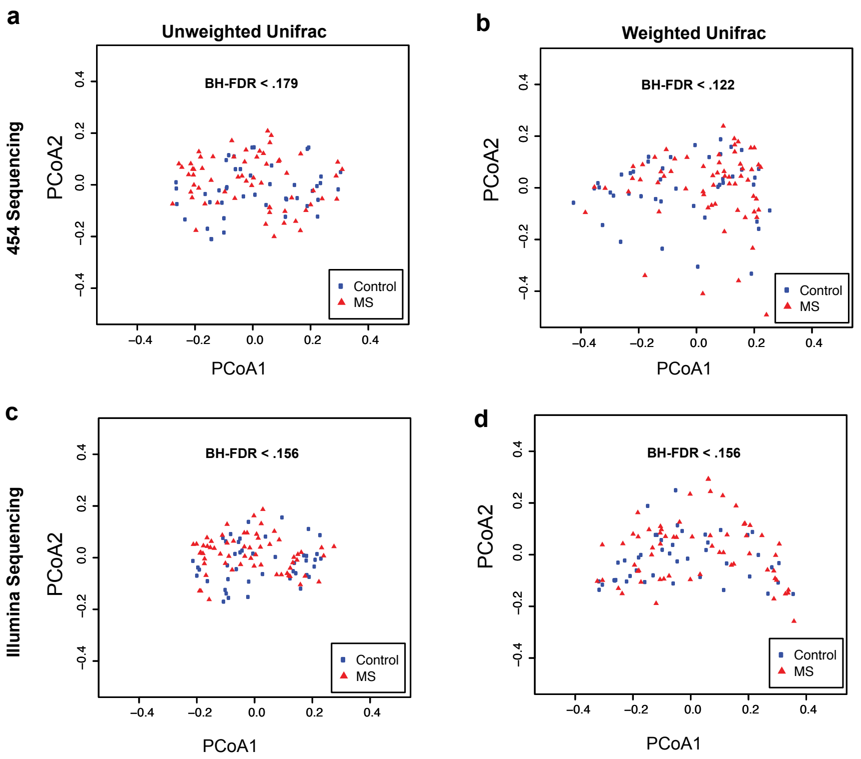

Supplementary Figure 3: Beta-diversity of the gut microbiota. Beta-diversity values were calculated using the UniFrac dissimilarity measure, to assess differences in overall microbial community structure. No significant differences were detected between MS patients (n = 60) and healthy controls (N = 43) using data from either the **a-b)** 454 Roche, or **c-d)** Illumina MiSeq platforms, and using either the unweighted (left) or weighted UniFrac (right) measures. The analysis of molecular variance (AMOVA) procedure was used for statistical hypothesis testing.

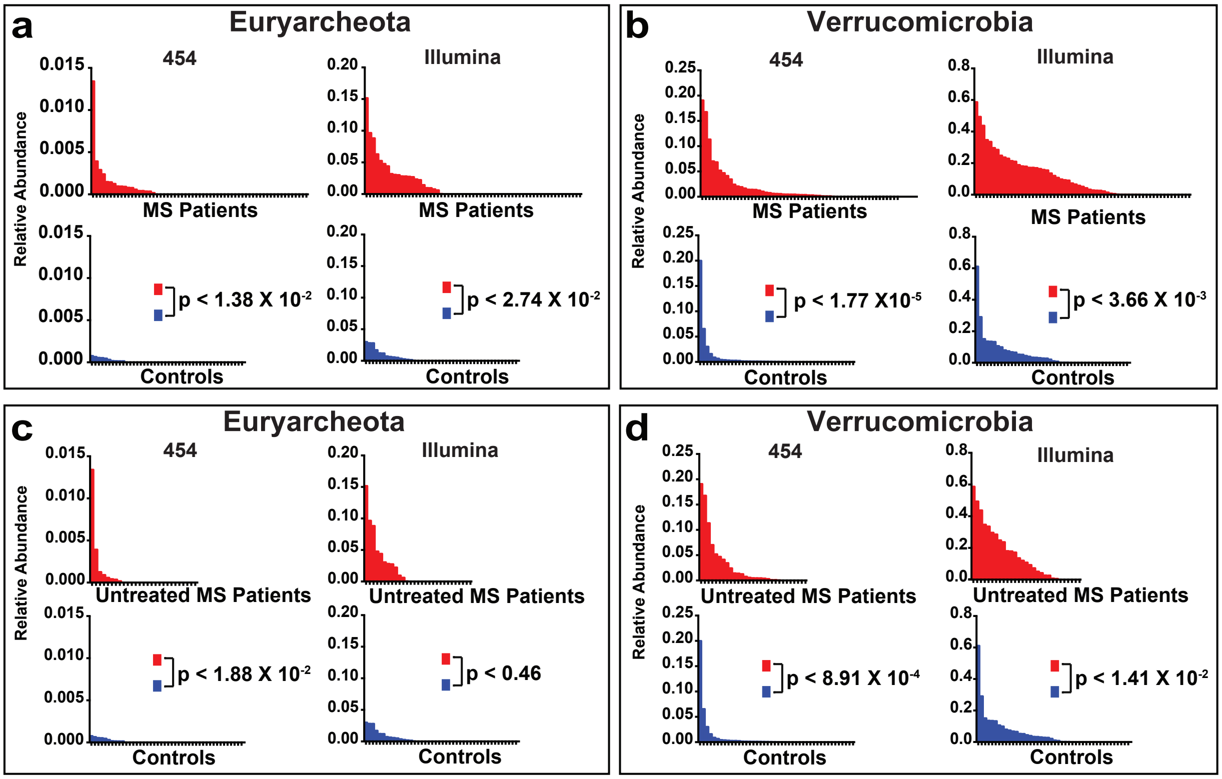

Supplementary Figure 4. Relative abundances of microbial phyla Euryarcheota and Verrucomicrobia in MS patients and controls. Panels **a,b**) compare all MS patients (n = 60) and controls (n = 43) while **c, d**) compare untreated MS patients (n = 28) and controls. Subjects are ordered along the x-axis according to the relative abundance (percentage of total sequencing reads) of the phylum beginning with samples with the highest abundances. Relative abundance of the phylum is depicted on the y-axis. P-values are adjusted for multiple hypothesis testing using the method of Benjamini-Hochberg.

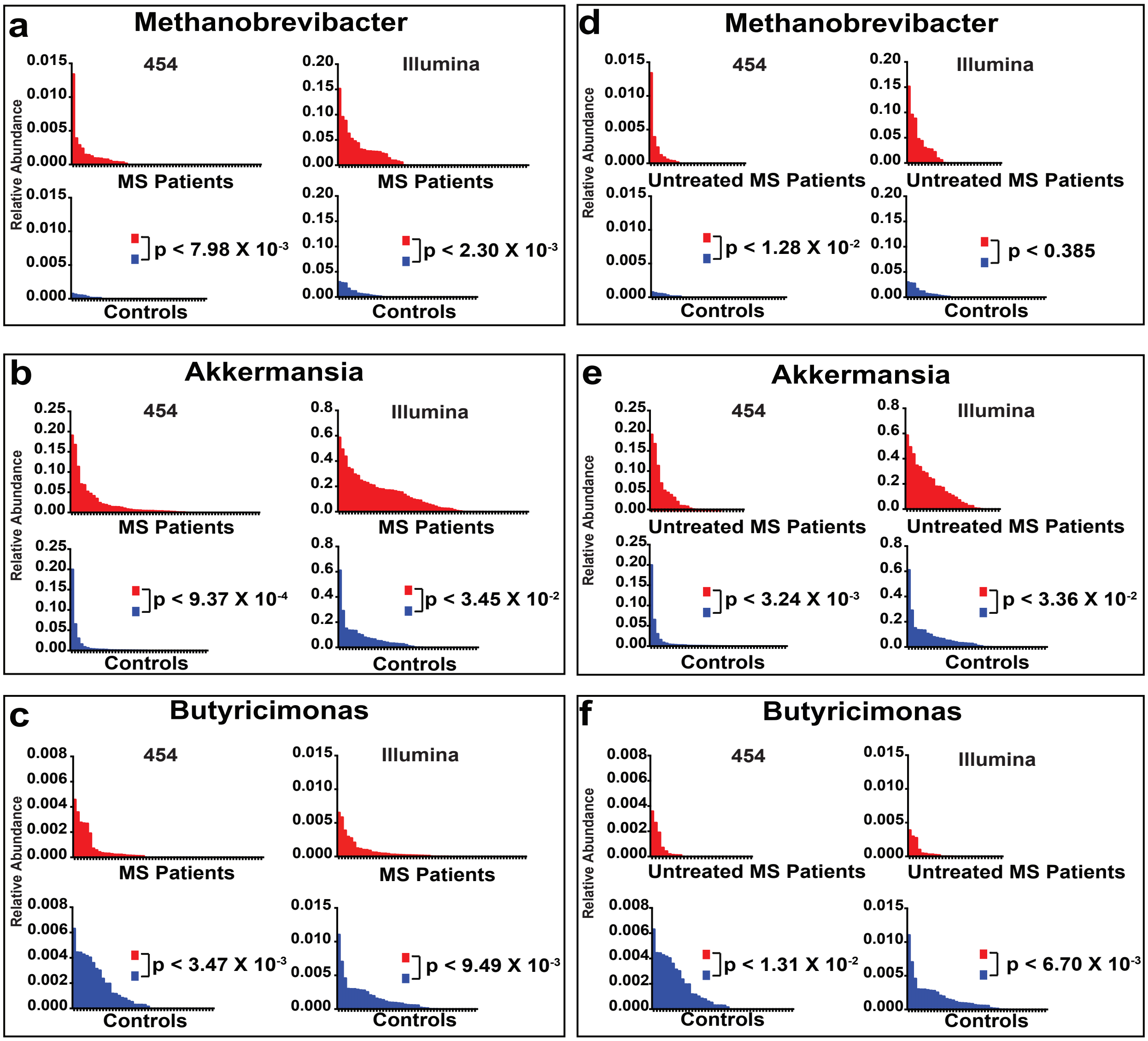

Supplementary Figure 5. Relative abundances of microbial genera Methanobrevibacter, Akkermansia, and Butyricimonas in MS patients and controls. Panels **a,b**) compare all MS patients (n = 60) and controls (n = 43) while **c, d**) compare untreated MS patients (n = 28) and controls. Subjects are ordered along the x-axis according to the relative abundance (percentage of total sequencing reads) of the genus beginning with samples with the highest abundances. Relative abundance of the genus is depicted on the y-axis. P-values are adjusted for multiple hypothesis testing using the method of Benjamini-Hochberg and a false discovery rate threshold of 0.1.

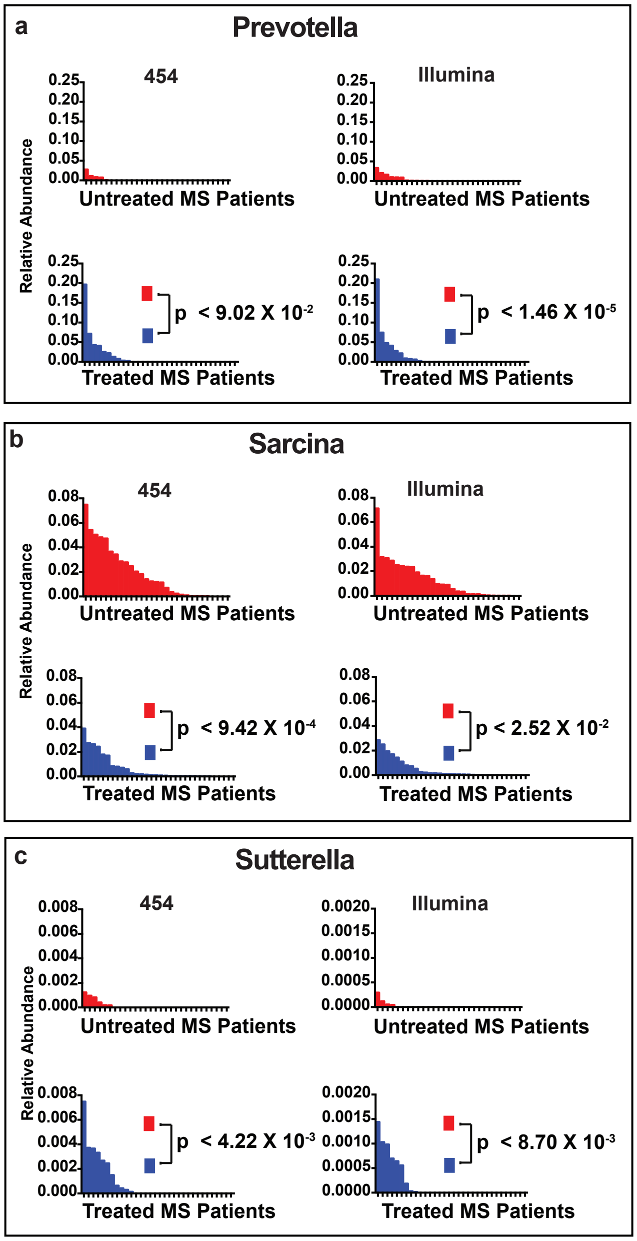

Supplementary Figure 6. Relative abundances of members of the microbial genera of Prevotella, Sutterella and Sarcina in untreated MS patients (n = 28) compared to treated MS patients (n = 32). Subjects are ordered along the x-axis according to the relative abundance (percentage of total sequencing reads) of the genus, beginning with samples with the highest abundances. Relative abundance of the genus is depicted on the y-axis. P-values are adjusted for multiple hypothesis testing using the method of Benjamini-Hochberg, and a false discovery rate threshold of 0.1.

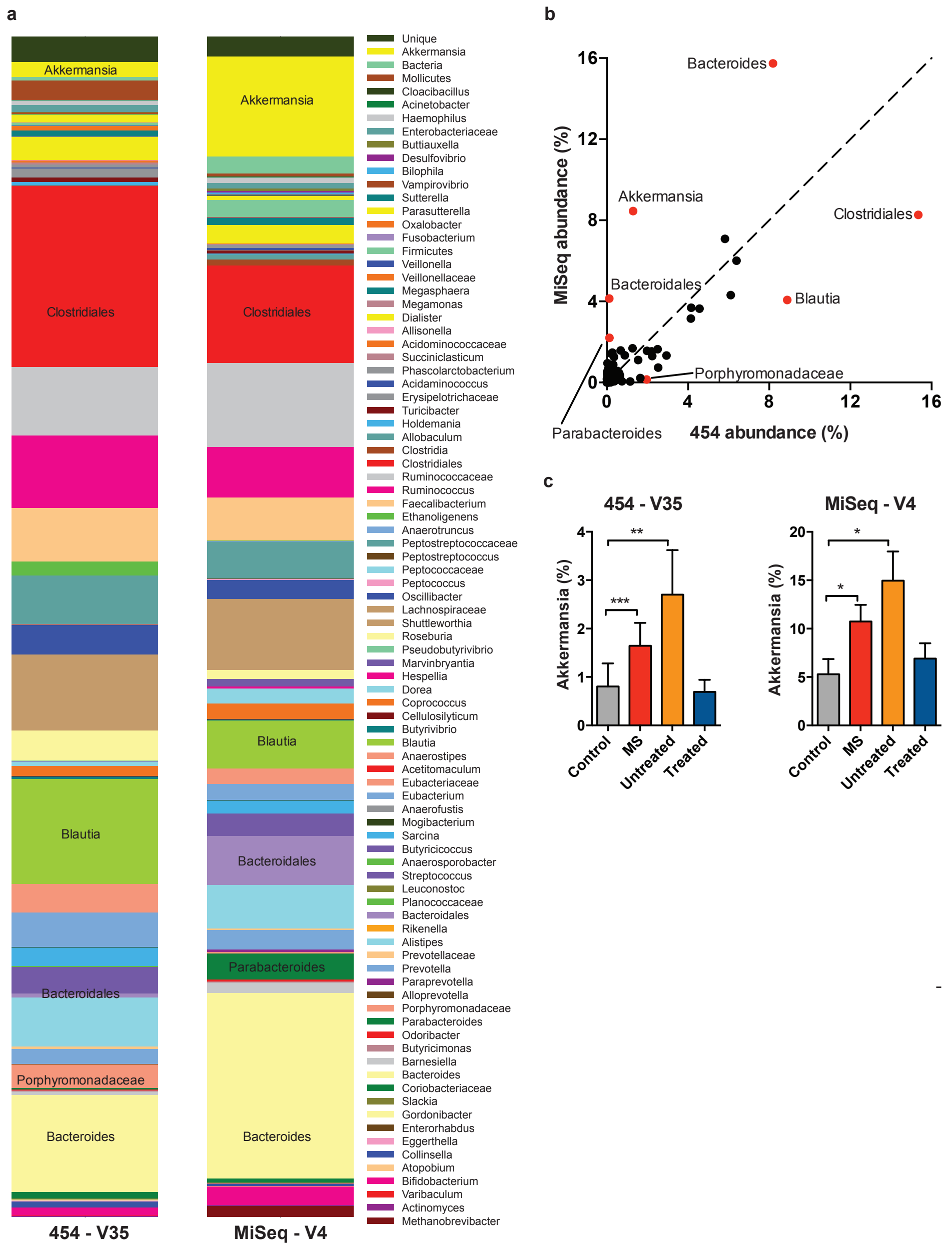

Supplementary Figure 7. Comparison of genera level differences based on sequencing technology. **a)** Relative abundance of shared taxa identified at the genus or lowest possible rank for 105 fecal microbiota samples sequenced with the microbial 16S rRNA V35 primer set using the Roche 454 platform or with the V4 primer set using the Illumina MiSeq platform. 98% of the microbial community was composed of shared genera, while approximately 2% of the total abundance was unique to either platform, indicating a large overlap of identified taxa. **b)** Comparison of the average microbial abdunance of the 90 shared genera detected on both the 454 or MiSeq platforms. The dashed line represents the line of identity (1 = 1). Example taxa that show marked differences in abdunance are highlighted in red and labeled. **c)** MS-related changes in Akkermansia are consistent between control and patient groups despite apparant primer biases between technology platforms. * $p < 0.05$, ** $p < 0.01$, *** $p < 0.001$, BH-adjusted DESeq.

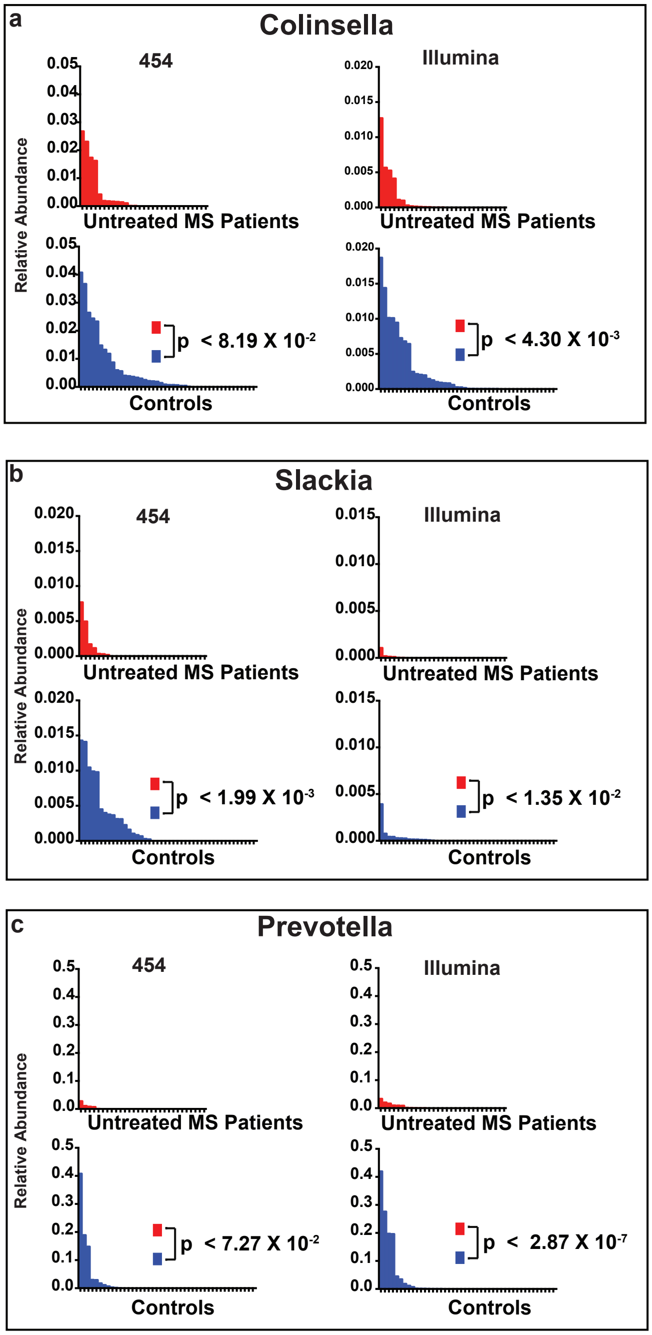

Supplementary Figure 8. Relative abundances of the microbial genera **a)** *Collinsella* **b)** *Slackia* and **c)** *Prevotella* in untreated MS patients (n = 28) and controls (n = 43). Patients are ordered along the x-axis according to the relative abundance (percentage of total sequencing reads) of the genus, beginning with samples with the highest abundances. Relative abundance of the genus is depicted on the y-axis. P-values were adjusted for multiple hypothesis testing using the method of Benjamini-Hochberg, and a false discovery rate threshold of 0.1 was used.

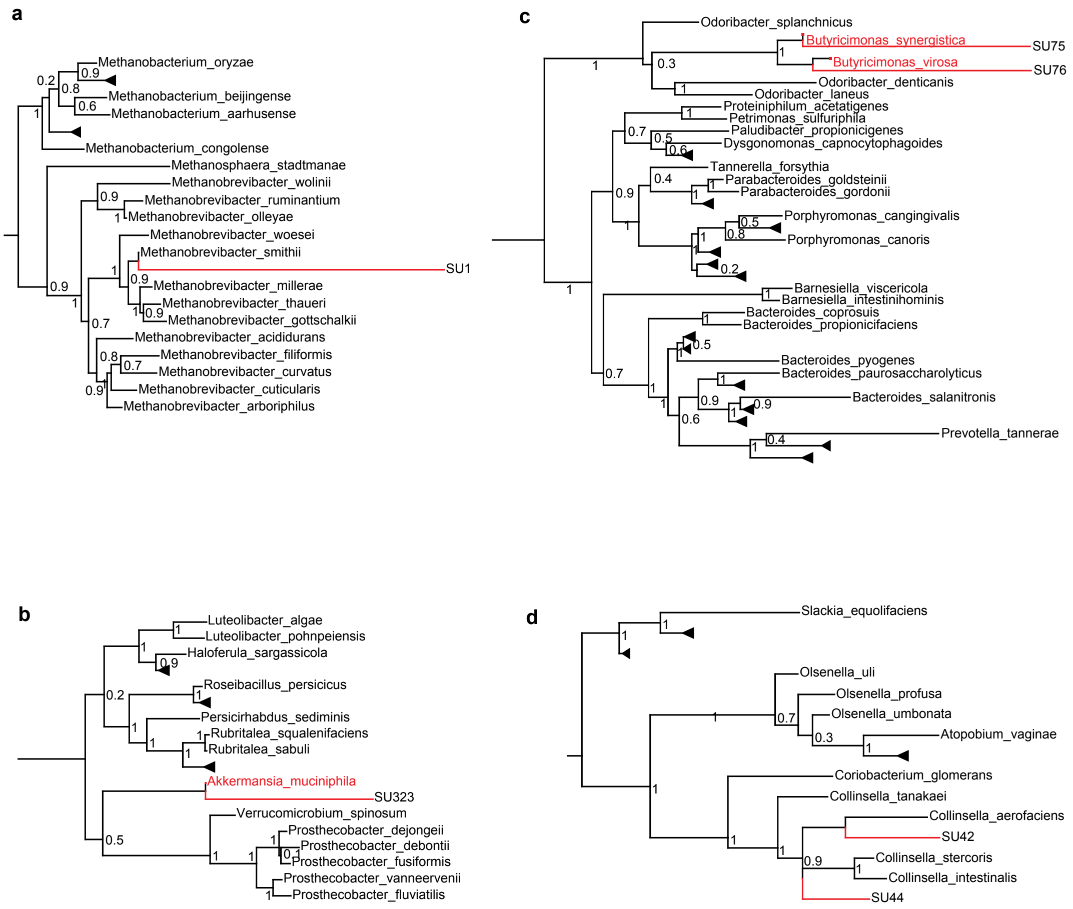

Supplementary Figure 9. Phylogenetic placement of representative unique sequences from differentially abundant genera in fecal samples from MS patients and controls. Placements are depicted for the genera: **a)** Methanobrevibacter, **b)** Akkermansia, **c)** Butyricimonas, **d)** Prevotella. 16S rRNA reference phylogenic trees were constructed using full-length of near full-length type strain sequences. Numbers on the reference tree branches are local support values, which are a measure of the reliability for a particular split on the tree (> 0.95 indicates strong support, > 0.70 indicates moderate support). Analyses indicate that for most of these genera, sequences could be resolved to one to two reference species.

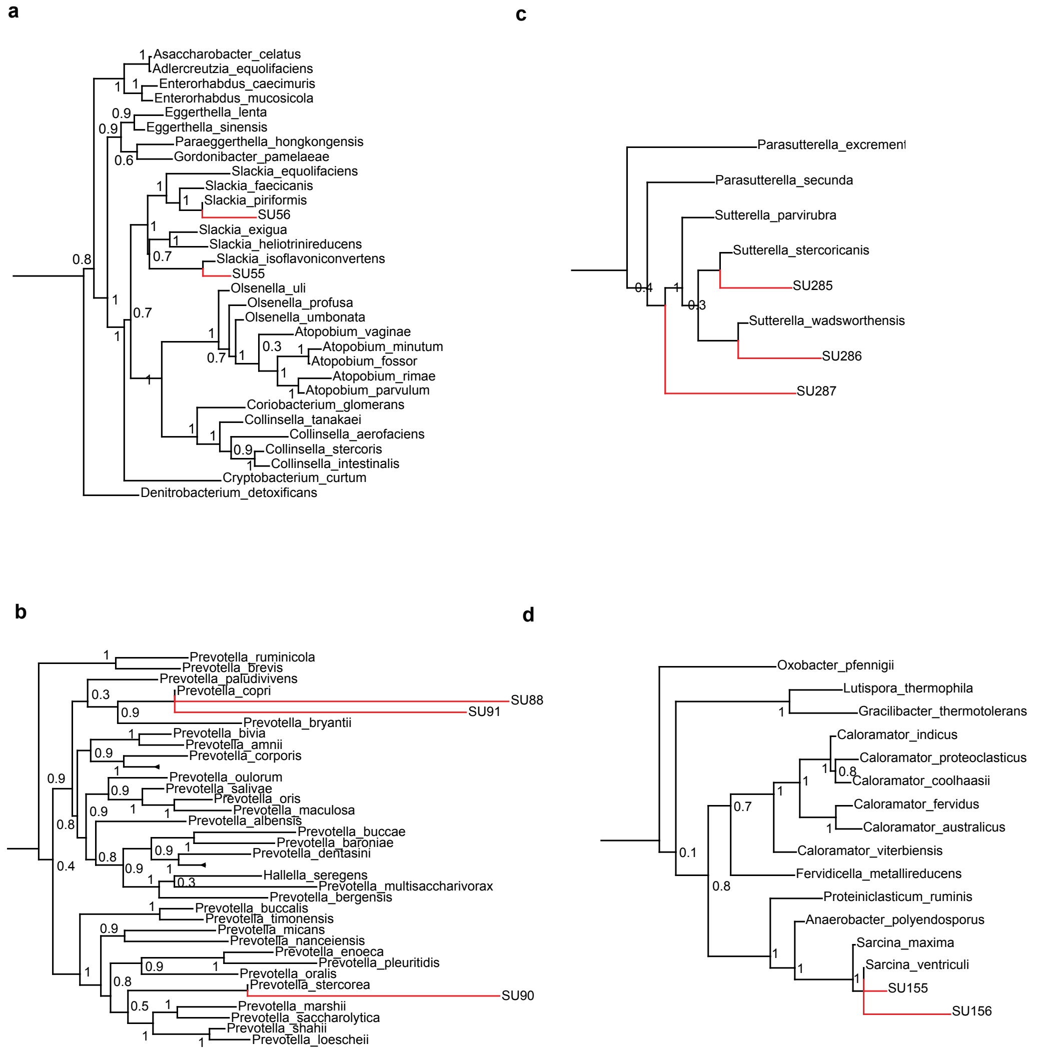

Supplementary Figure 10. Phylogenetic placement of representative unique sequences from differentially abundant genera in fecal samples from MS patients and controls. Placements are depicted for the genera: **a)** Collinsella, **b)** Slackia, **c)** Sutterella, and **d)** Sarcina. 16S rRNA reference phylogenic trees were constructed using full-length of near full-length type strain sequences. Numbers on the reference tree branches are local support values, which are a measure of the reliability for a particular split on the tree (> 0.95 indicates strong support, > 0.70 indicates moderate support). Analyses indicate that for most of these genera, sequences could be resolved to one to two reference species.

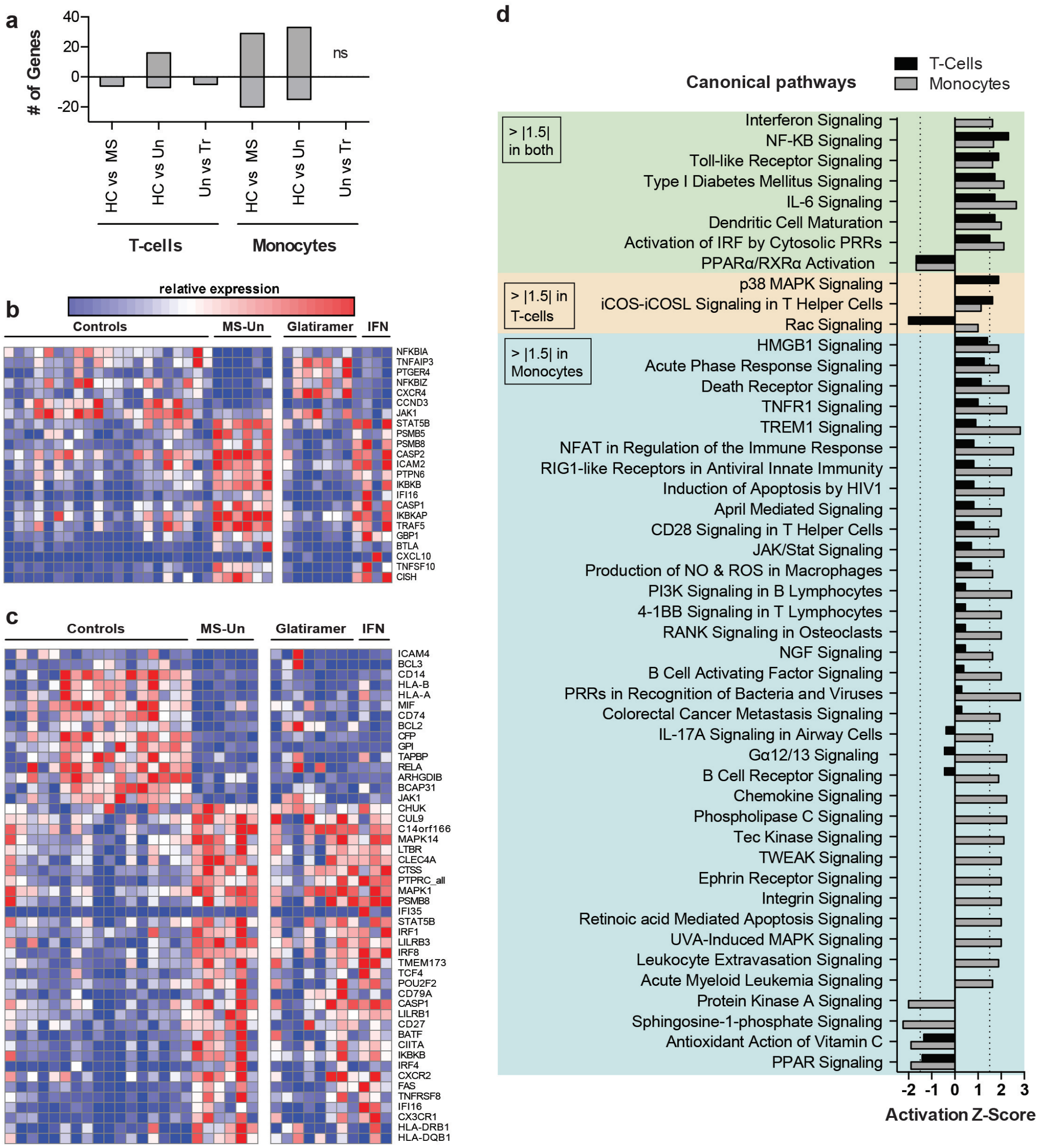

Supplementary Figure 11. Alterations in the transcriptome of monocytes and T cells from MS patients and controls. a) Number of genes significantly increased or decreased in T cells and monocytes in control (n = 20 T cells, 17 monocytes) vs. all MS patients (n = 18 T cells, 17 monocytes), control vs. untreated-MS patients (n = 7 T cells, 6 monocytes) (disease effect), and untreated vs treated MS patients (n = 11 T cells, 11 monocytes) (treatment effect). **b-c)** Expression of genes that are significantly altered between controls and untreated MS patients in T cells **b)** and **c)** monocytes. **d)** Canonical pathways significantly altered in untreated MS patients compared to controls. Pathways that have an activation Z-score > |1.5| are listed.

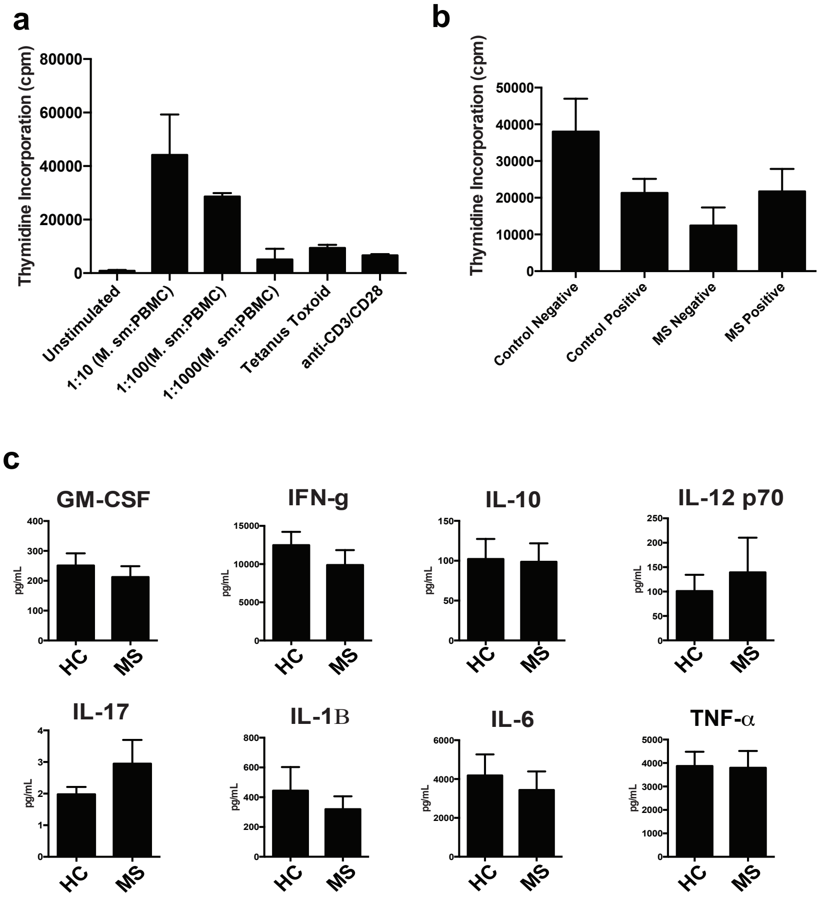

Supplementary Figure 12: Proliferative responses and cytokine production in PBMCs from MS and controls following culture with *Methanobrevibacter smithii*. **a)** Stimulation of PBMCs with *Methanobrevibacter smithii*, tetanus toxoid or anti-CD3/CD28 in 15 healthy donors. *Methanobrevibacter smithii* was cultured in a ratio of 1 bacteria to 1, 10, 100, or 1000 PBMCs. Thymidine incorporation was measured 4 days following stimulation. **b)** *Methanobrevibacter smithii* stimulation of subjects positive or negative for detectable *Methanobrevibacter smithii* in the gut (7 HC+, 8 HC-, 8 MS+, 6 MS-). **c)** Cytokine production as measured by Luminex comparing HC (n = 15) and MS patients (n=14) following stimulation of PBMCs by *Methanobrevibacter smithii*.

Supplementary Table 1: Demographics of Subset of Multiple Sclerosis Patients and Healthy Subjects Utilized for T cell and Monocyte Assays

Groups	n	Age	Gender	BMI[a]	Medications	EDSS[b] Score	Disease Duration
MS Patients with recoverable Methano-brevibacter	10	52.2	7 F[c]<br>3 M[c]	26.2	4 untreated<br>2 interferon<br>4 glatiramer acetate	1.5	18.5±6.8
MS Patients with no recoverable Methanobrevibacter	8	43.8	5 F<br>3 M	28.3	3 untreated, 1 interferon, 4 glatiramer acetate	1.2	12.75±5.2
All MS in Subset	18	48.4	12 F<br>6 M	27.1	7 untreated<br>3 interferon<br>8 glatiramer acetate	1.4	15.95±6.7
Healthy Subjects with recoverable Methano-brevibacter	10	41.3	6 F<br>4 M	27.7			
Healthy Subjects with no recoverable Methanobrevibacter	8	39.5	6 F<br>2 M	25.9			
All Healthy Subjects in Subset	18	40.5	12 F<br>6 M	26.9			

[a]BMI: Body mass index
[b]EDSS: Expanded disability status score
[c]F: Female, M: Male

Supplementary Table 2: Demographics of MS patients and controls recruited for methane breath test

	Multiple Sclerosis	Healthy Controls
Total Number	41	32
Mean Age	44	36
Males	9	12
Females	31	20
Relapsing-Remitting Untreated	10	-
Relapsing-Remitting Glatiramer Acetate	13	-
Relapsing-Remitting Interferon	18	-

Supplementary Table 3: Dietary History

	Healthy	Multiple Sclerosis	p-value (Fisher Exact Test)
History of Vegetarian Diet	9 (21)	9 (14.8)	.44
Milk Consumption (%)			.03
High	6 (14)	19 (31.1)	
Low	37 (86)	40 (65.6)	
Yogurt Consumption (%)			.20
High	7 (16.3)	4 (6.6)	
Low	36 (83.7)	52 (82.5)	
Fast Food (%)			.80
High	9 (20.9)	10 (16.4)	
Low	34 (79.1)	45 (71.4)	
